# Small Extracellular Vesicles From Human Amniotic Membrane Mesenchymal Stem Cells Rejuvenate Senescent β Cells and Cure Age‐Related Diabetes in Mice

**DOI:** 10.1111/acel.70327

**Published:** 2025-12-14

**Authors:** Lei Xiao, Zicheng Zhang, Tong Li, Yuyin Jiang, Yuanxin Liu, Tingting Lv, Lianju Qin, Yunxia Zhu, Wei Tang

**Affiliations:** ^1^ Department of Endocrinology Geriatric Hospital of Nanjing Medical University Nanjing Jiangsu China; ^2^ Key Laboratory of Human Functional Genomics of Jiangsu Province, Department of Biochemistry and Molecular Biology Nanjing Medical University Nanjing Jiangsu China; ^3^ State Key Laboratory of Reproductive Medicine, Center of Clinical Reproductive Medicine, First Affiliated Hospital Nanjing Medical University Nanjing China

**Keywords:** β‐cell senescence, age‐related diabetes, human amniotic membrane mesenchymal stem cell, miR‐21‐5p, mitochondrial calcium homeostasis, small extracellular vesicles

## Abstract

Targeting senescent pancreatic β‐cells represents a promising therapeutic avenue for age‐related diabetes; however, current anti‐senescence strategies often compromise β‐cell mass. In this study, human amniotic mesenchymal stem cell‐derived small extracellular vesicles (hAMSC‐sEVs) were identified as a novel intervention that can be used to effectively counteract cellular senescence and preserve β‐cell integrity. We aimed to systemically delineate the molecular mechanisms underlying hAMSC‐sEV‐mediated reversal of β‐cell senescence in age‐related diabetes. In oxidative stress‐induced and naturally aged β‐cell models, hAMSC‐sEVs mitigated senescence‐associated phenotypes, restored mitochondrial homeostasis, and enhanced insulin secretion capacity. In aged diabetic mice, administering these vesicles significantly ameliorated hyperglycemia, improved glucose tolerance, and reversed β‐cell functional decline by reducing senescent β‐cell populations, reinstating β‐cell identity markers, and suppressing senescence‐associated secretory phenotype (SASP) component production. Mechanistic investigations revealed that the miR‐21‐5p‐enriched hAMSC‐sEVs directly target the interleukin (IL)‐6 receptor α subunit (IL‐6RA), thereby inhibiting signal transducer and activator of transcription 3 (STAT3) phosphorylation at tyrosine 705 and its subsequent nuclear translocation. This epigenetic modulation alleviated STAT3‐mediated transcriptional repression of the mitochondrial calcium uniporter (MCU), rectifying age‐related mitochondrial calcium mishandling and insulin secretion defects. Genetic ablation of MCU clearly established the central role of the miR‐21‐5p/IL‐6RA/STAT3/MCU axis in this regulatory cascade. Our findings reveal hAMSC‐sEVs as a novel senotherapeutic strategy for age‐related diabetes, elucidating the pivotal role of miR‐21‐5p‐driven epigenetic–mitochondrial calcium homeostasis in reversing β‐cell dysfunction, establishing a framework for targeting cellular senescence in metabolic disorders.

## Graphical abstract

hAMSC‐sEVs rejuvenate β cells and improve glycaemic control via the miR‐21‐5p/IL‐6RA/STAT3/MCU axis, restoring mitochondrial Ca^2+^ homeostasis and insulin secretion in aged T2DM mice.

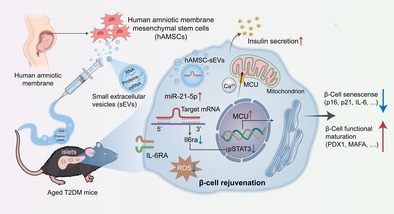

**Abbreviations**
γ‐H2AX: phosphorylated histone H2AX on serine 139
ΔΨm: mitochondrial membrane potential
AKT: protein kinase B
ATP: adenosine triphosphate
CCL2: C‐C motif chemokine ligand 2
ChIP: chromatin immunoprecipitation
CUT and Tag: Cleavage Under Targets and Tagmentation
EDU: 5‐Ethynyl‐2′‐deoxyuridine
GO enrichment: gene ontology enrichment analysis
GSIS: glucose‐stimulated insulin secretion
H_2_O_2_
: hydrogen peroxide
hAMSC: human amniotic mesenchymal stem cell
HFD: high‐fat diet
IL‐1β: interleukin‐1 beta
IL‐6: interleukin‐6
IL‐6RA: IL‐6 receptor α subunit
Ins1: insulin1
IPGTTs: intraperitoneal glucose tolerance tests
IPITTs: intraperitoneal insulin tolerance tests
IRS1: insulin receptor substrate 1
KEGG enrichment: Kyoto Encyclopedia of Genes and Genomes pathway enrichment analysis
Mafa: musculoaponeurotic fibrosarcoma homolog A
MCU: mitochondrial calcium uniporter
MIN6: mouse insulinoma beta cell line
miRNA: micro ribonucleic acid
mtCa^2+^
: mitochondrial calcium
mtSOX: mitochondrial superoxide
NCD: normal chow diet
OCR: oxygen consumption rate
PBS: phosphate buffer saline
Pdx1: pancreatic and duodenal homeobox 1
qPCR: quantitative real‐time polymerase chain reaction
ROS: reactive oxygen species
SASP: senescence‐associated secretory phenotype
SA‐β‐Gal: senescence‐associated β‐galactosidase
sEVs: small extracellular vesicles
Slc2a2: solute carrier family 2 member 2
STAT3: signal transducer and activator of transcription 3
STZ: streptozotocin
T2DM: type 2 diabetes mellitus
TEM: transmission electron microscopy
TNF‐α: tumor necrosis factor alpha
TSS: transcription start sites

## 1 Introduction

Aging represents a progressive decline in physiological and metabolic homeostasis, resulting in systemic tissue degeneration and death of organisms (Lopez‐Otin et al. 2023). The increasing geriatric population has positioned age‐related pathologies, particularly type 2 diabetes mellitus (T2DM), cardiovascular disorders, neurodegenerative diseases, and malignancies, as primary contributors to global morbidity, healthcare costs, and mortality rates (GBD 2021 Diseases and Injuries Collaborators 2024). This urgency drives scientific exploration of fundamental aging mechanisms and the development of targeted anti‐aging therapeutics. Mechanistically, aging results from the interplay of multiple interconnected mechanisms: cellular senescence, stem cell exhaustion, aberrant intercellular communication, mitochondrial dysfunction, genomic instability, telomeric dysfunction, epigenetic dysregulation, proteostatic collapse, and metabolic signaling disruption. Cellular senescence, a stress‐induced irreversible growth arrest, plays a central role in orchestrating aging pathophysiology (Lopez‐Otin et al. 2023). Considering the complex biological aging mechanisms, the scientific community is developing intervention strategies targeting distinct aging pathways, with senolytic therapies (Amor et al. 2020; Haston et al. 2023), cellular reprogramming technologies (Browder et al. 2022; Jing et al. 2023), heterochronic parabiosis models (Ma et al. 2022; Zhang et al. 2023), stem cell‐based regenerative medicine approaches (Zhu et al. 2021, 2024), and dietary interventions for metabolic regulation (Acosta‐Rodriguez et al. 2022; Duregon et al. 2023) emerging as the most promising anti‐aging strategies.

T2DM is characterized by insulin resistance and β‐cell dysfunction. It drives diabetes‐specific complications (cardiovascular disease, nephropathy, retinopathy, and neuropathy) (American Diabetes Association 2021) and exacerbates age‐related pathologies by systemically inducing senescence pathways (Blazer et al. 2002; Kitada et al. 2014; Shosha et al. 2018). Evidence reveals that progressive β‐cell senescence correlates with aging, elevated body mass index, and insulin resistance progression (Aguayo‐Mazzucato et al. 2019; Yu et al. 2020). These senescent β‐cells display a hallmark of features, including elevated senescence‐associated β‐galactosidase (SA‐β‐Gal) activity, p16 upregulation, Lamin B1 suppression, and secretion of senescence‐associated secretory phenotype (SASP) components, such as interleukin (IL)‐1β, IL‐6, and C‐C motif chemokine ligand 2 (CCL2) (Midha et al. 2021). Through paracrine signaling, SASP mediators induce islet microenvironment destabilization and promote insulitis (Talchai et al. 2012; Midha et al. 2021; Lopez‐Otin et al. 2023). Notably, senescent cells are resistant to apoptosis, favoring the preferential survival of aged β‐cells under metabolic stress, thereby resulting in cellular persistence, amplified SASP propagation, β‐cell dedifferentiation, functional decline, and glucose dysregulation. Findings from our previous studies revealed that islet‐derived inflammation disseminates via proinflammatory macrophages and extracellular vesicles (EVs) enriched with miR‐29 and miR‐503 to insulin‐responsive tissues (liver, skeletal muscle, adipose tissue), which establishes a feed‐forward cycle of peripheral inflammation and insulin resistance—a critical pathway in geriatric T2DM pathogenesis (Sun et al. 2021; Zhou et al. 2024). Thus, β‐cell senescence contributes to T2DM pathophysiology through secretory failure and insulin resistance potentiation, establishing it as a pivotal mediator in diabetes progression among older adults.

Emerging senotherapeutic strategies specifically targeting senescent β‐cells show therapeutic potential. Notably, the primary islet β‐cells in mice respond to senolytic agents (ABT263, ABT199, quercetin + dasatinib) (Aguayo‐Mazzucato et al. 2019; Thompson et al. 2019). Clearance of these β‐cells alleviates obesity‐, insulin receptor antagonist‐, and aging‐induced glucose intolerance in mice, restoring β‐cell function and identity (Aguayo‐Mazzucato et al. 2019). However, in these studies, safety concerns, including β‐cell mass reduction (Aguayo‐Mazzucato et al. 2019; Thompson et al. 2019), compromised tissue repair mechanisms, potential carcinogenicity, and non‐specific cellular targeting are highlighted regarding senolytic compounds (Gasek et al. 2021; Murakami et al. 2022). Thus, safer and more effective anti‐senescence strategies that preserve β‐cell integrity are needed. Recent evidence shows that small EVs (sEVs) derived from embryonic stem cells (Yu et al. 2023; Bi et al. 2025) and juvenile mesenchymal stem cells (Dorronsoro et al. 2021; Sanz‐Ros et al. 2022, 2025) can reverse cellular senescence, ameliorate functional decline in muscle, kidney, liver, and heart, and extend lifespan. Their therapeutic effects are mechanistically associated with the bioactive payload of sEVs, which contains senescence‐regulating microRNAs (miRNAs) and functional protein cargos (Sanz‐Ros et al. 2022; Yu et al. 2023; Bi et al. 2025). Investigations exploring stem cell‐derived sEVs for reversing β‐cell senescence in age‐associated diabetes pathogenesis are notably absent from current research paradigms. Human amniotic mesenchymal stem cells (hAMSCs), isolated from clinically discarded fetal membranes, provide an ethically acceptable source of multipotent stromal cells with distinct therapeutic advantages (Kulus et al. 2021). Compared with those of conventional stem cell sources, hAMSCs exhibit superior clinical translatability owing to their non‐invasive isolation protocols, absence of teratogenic potential, and robust paracrine bioactive secretion (Liu, Huang, et al. 2021; Liu, Jiang, et al. 2021). sEVs, which are 30–200 nm phospholipid vesicles actively secreted by cells, serve as critical mediators of intercellular communication through targeted delivery of functional miRNAs, proteins, and lipid cargos (Dixson et al. 2023). Unlike whole‐cell transplantation, sEV‐based therapeutics preserve progenitor cell efficacy and circumvent critical limitations, including alloimmune responses and tumorigenic risks (Tan et al. 2024). We present a pioneering β‐cell rejuvenation paradigm leveraging hAMSC‐sEVs as senescence‐modulating nanotherapeutics. In this investigation, the molecular mechanisms underlying hAMSC‐sEV‐mediated reversal of β‐cell senescence in age‐related diabetes were systematically delineated. Our findings reveal that hAMSC‐sEVs undergo efficient β‐cell internalization, delivering senescence‐regulating miRNA payloads. Mechanistically decoded, miR‐21‐5p emerges as a key regulator of β‐cell rejuvenation through coordinated modulation of the IL‐6RA/STAT3/MCU axis, restoring mitochondrial calcium homeostasis and insulin secretory competence.

## 2 Results

### 2.1 hAMSC‐sEVs Ameliorate β‐Cell Senescence In Vitro

The isolated hAMSCs met international immunophenotypic criteria with ≥ 98% positivity for CD73/CD90/CD105 and ≤ 2% expression of hematopoietic lineage markers (CD11b/CD19/CD34/CD45/HLA‐DR). The stem cells also maintained multilineage differentiation capacity toward osteogenic, adipogenic, and chondrogenic lineages (Figure S1a,b). Ultracentrifugation‐purified sEVs (Figure S1c) were biochemically authenticated based on triple validation: (1) Exosome‐specific protein signature (CD9+/CD63+/TSG101+/calnexin–) using immunoblotting (Figure S1d), (2) characteristic cup‐shaped ultrastructure via transmission electron microscopy (TEM) (Figure S1e), and (3) nanoparticle tracking, confirming 91.2% of vesicles within the 80–200 nm range (mode: 118.6 nm) (Figure S1f).

Hydrogen peroxide (H_2_O_2_) has been proven to induce premature senescence of β‐cells by increasing oxidative stress (Aguayo‐Mazzucato et al. 2019; Song et al. 2022). We established a controlled senescence model in MIN6 cells through a 2‐h oxidative pulse (200 μM H_2_O_2_) followed by a 48‐h recovery in fresh medium to investigate the anti‐senescence effects of hAMSC‐sEVs (Figure S2a). The established senescence model triggered β‐cell pathophysiological remodeling through three‐phase alterations: initial oxidative stress induced robust SA‐β‐gal activation with phosphorylated histone H2AX on serine 139 (γ‐H2AX) foci accumulation (Figure S2b–e), followed by molecular reprogramming confirmed by cell cycle inhibitors (p16, p21) and SASP activation (*Il1β*, *Il6*, *Ccl2*) (Figure S2f–i). Progressive functional decompensation was characterized by insulin secretion reduction (Figure S2k,l), accompanied by β‐cell maturation regulator suppression (insulin [*Ins1*], musculoaponeurotic fibrosarcoma homolog a [*Mafa*], pancreatic and duodenal homeobox 1 [*Pdx1*], solute carrier family 2 member 2 [*Slc2a2*]) (Figure S2j), ultimately demonstrating a temporal correlation between persistent DNA damage and the progression of insulin deficiency (Figure S2m,n).

**FIGURE 1:**
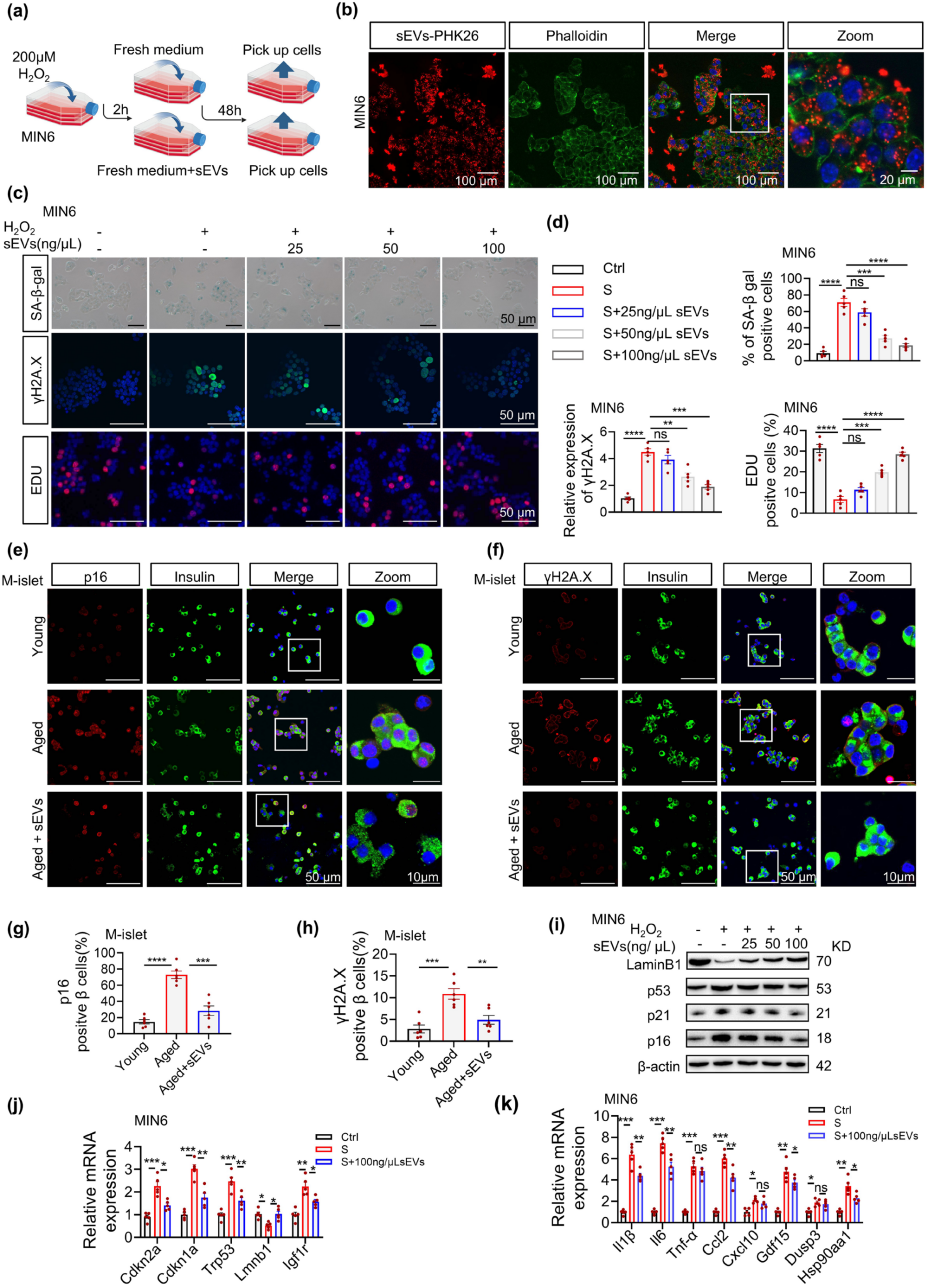
hAMSC‐sEVs ameliorate β‐cell senescence in vitro. (a–d) sEV intervention in H_2_O_2_‐induced senescence in MIN6 cells. (a) Experimental timeline: cells are pretreated with H_2_O_2_ (200 μM, 2 h) with/without sEVs (25–100 ng/μL, 48 h). (b) PKH26‐labeled sEV uptake is shown (red) after 24 h. Scale bars: 100 μm (overview panels); 20μm (oom). (c) Senescence marker staining shows SA‐β‐gal (blue), γ‐H2AX foci (green), and EdU^+^ proliferative cells (red). Scale bars, 50 μm. (d) Quantification shows SA‐β‐gal^+^ cells (%), γ‐H2AX intensity, and EdU^+^ cells (%); *n* = 5 per group. (e–h) sEV intervention in aging‐associated senescence in C57BL/6J islets from young (2‐month), aged (18‐month), and aged + sEVs (100 ng/μL, 48 h) groups: (e) p16 (red)/insulin (green) co‐staining is shown. Scale bars: 50 μm (overview panels); 10 μm (Zoom). (f) γ‐H2AX (red)/insulin (green) co‐staining is shown. Scale bars: 50 μm (overview panels); 10 μm (Zoom). (g, h) Quantification shows p16^+^ β‐cells (%) (g) and γ‐H2AX^+^ β‐cells (%) (h); *n* = 6 per group. (i–k) Molecular profiling. (i) Western blots show senescence markers (Lamin B1, p53, p21, p16). (j) qPCR shows senescence‐related mRNAs (*Cdkn2a, Cdkn1a, Trp53, Lmnb1, Igf1r*); *n* = 5 per group. (k) qPCR shows SASP mRNAs (*Il1b, Il6, Tnf, Ccl2, Cxcl10, Gdf15, Dusp3, Hsp90aa1*); *n* = 5 per group. Each dot represents one independent experiment; data are presented as mean ± SEM. *p < 0.05, **p < 0.01, ***p < 0.001, ***p < 0.0001; ns, not significant.

After co‐incubating senescent MIN6 cells with hAMSC‐sEVs (25–100 ng/μL, Figure 1a), PKH26‐labeled hAMSC‐sEVs showed progressive internalization, with substantial accumulation observed in MIN6 cells at 24 h (Figure 1b). hAMSC‐sEV administration significantly reduced the SA‐β‐gal‐positive cell proportion, ameliorated the senescence‐associated DNA damage response, and restored proliferative capacity, confirmed by an increase in 5‐Ethynyl‐2′‐deoxyuridine (EdU)‐positive cells (Figure 1c,d). The anti‐senescence effects were further validated in primary islets from naturally aged mice, demonstrating reduced p16‐positive and γ‐H2AX‐positive β‐cell populations (Figure 1e–h). Western blotting revealed hAMSC‐sEV‐mediated suppression of p16/p21/p53 proteins with concurrent LaminB1 upregulation (Figure 1i), paralleled by corresponding mRNA expression changes (Figure 1j). hAMSC‐sEVs at a 100 ng/μL concentration potently inhibited SASP‐associated genes (*Il1β*, *Il6*, *Ccl2*) in senescent β‐cells (Figure 1k). Collectively, these findings show that these stem cells could reverse β‐cell senescence induced by oxidative stress and natural aging.

### 2.2 hAMSC‐sEVs Rescue Senescent β‐Cell Functions

**FIGURE 2:**
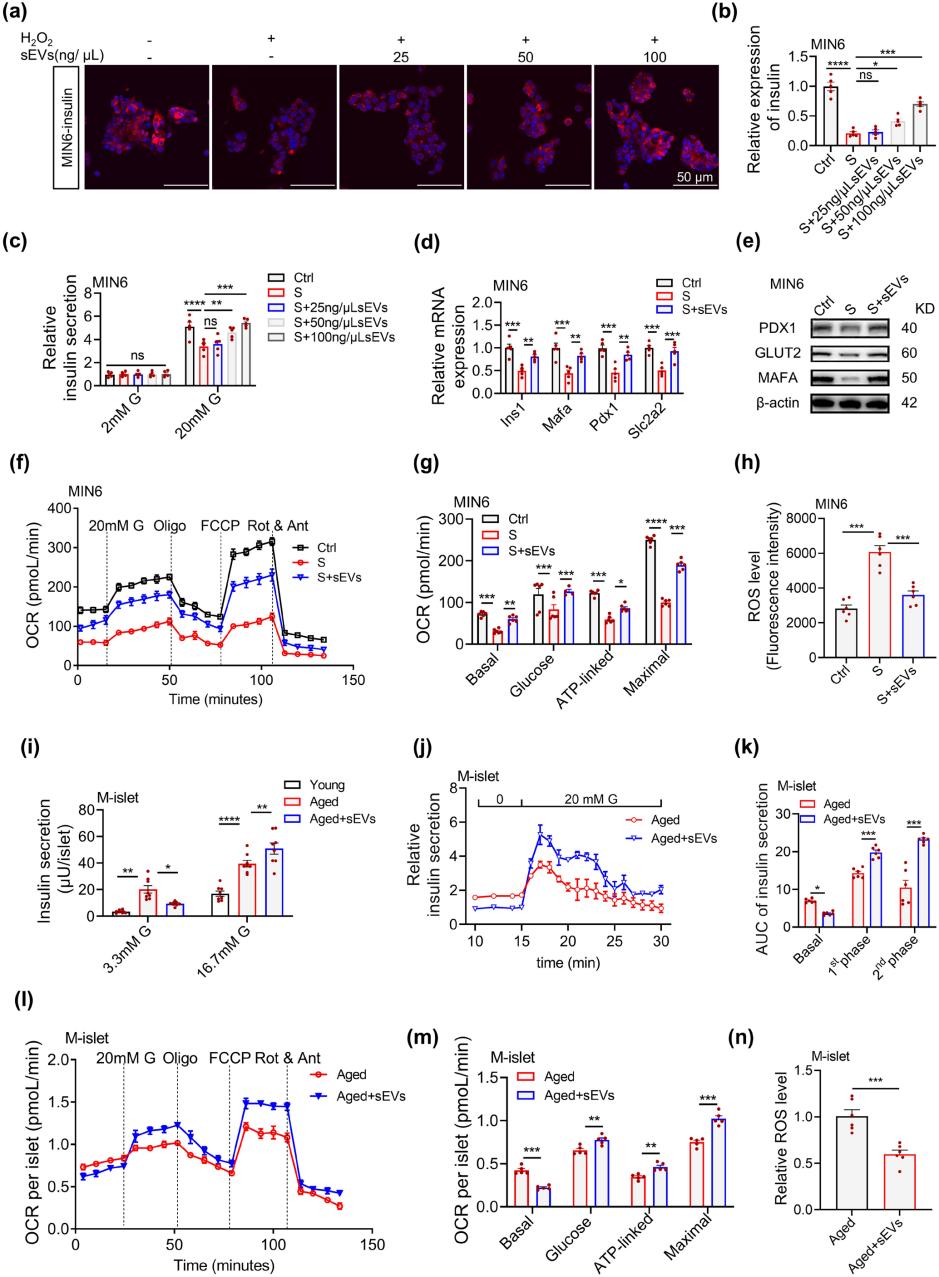
hAMSC‐sEVs restore insulin secretion and mitochondrial metabolic homeostasis in senescent β‐cells. (a–e) sEV intervention in H_2_O_2_‐induced senescence in MIN6 cells; cells are pretreated with H_2_O_2_ (200 μM, 2 h) with/without sEVs (25–100 ng/μL, 48 h). (a) Insulin immunofluorescence (red) is shown. Scale bars, 50 μm. (b) sEVs dose‐dependently enhance insulin content; *n* = 5 per group. (c) GSIS profile is restored; *n* = 5 per group. (d, e) β‐cell maturation markers (*Ins1, Mafa, Pdx1, Slc2a2*) are upregulated at the mRNA (d) and protein (e) levels. (f–h) Oxygen consumption rate (OCR) analysis (f, g) and ROS levels (h) in MIN6 cells under three conditions: Control (Ctrl), senescent (S), and senescent + sEVs (100 ng/μL; S + sEVs); *n* = 6 per group. (i) Insulin secretion in C57BL/6J islets from young (2‐month), aged (18‐month), and aged + sEVs (100 ng/μL, 48 h) groups under low (3.3 mM) versus high (16.7 mM) glucose. (j, k) Islet perifusion of islets from 18‐month C57BL/6J mice treated with sEVs (100 ng/μL) or vehicle for 48 h (j); AUC is analyzed across phases: Basal (10–15 min), first phase (15–20 min), and second phase (20–30 min) (k). (l–n) OCR analysis (l, m) and ROS levels (n) in C57BL/6J islets from aged (18‐month) and aged + sEVs (100 ng/μL, 48 h) groups; *n* = 5–6 per group. Each dot represents one independent experiment; data are presented as mean ± SEM. *p < 0.05, **p < 0.01, ***p < 0.001, ****p < 0.0001; ns, not significant.

Next, we investigated the effects of hAMSC‐sEVs on β‐cell function. Insulin immunofluorescence staining and glucose‐stimulated insulin secretion (GSIS) assays showed that hAMSC‐sEVs dose‐dependently enhanced insulin synthesis in senescent MIN6 cells (Figure 2a,b) and improved high GSIS (Figure 2c). The restored expression of β‐cell functional maturity markers (*Ins1*, *Mafa*, *Pdx1*, *Slc2a2*) at mRNA and protein levels showed that hAMSC‐sEVs may reverse β‐cell senescence (Figure 2d,e). Given the crucial role of mitochondrial oxidative phosphorylation in insulin secretion and its documented decline during β‐cell aging (Cree et al. 2008; Gregg et al. 2016), we assessed mitochondrial respiration and reactive oxygen species (ROS) levels. hAMSC‐sEVs significantly ameliorated the impaired mitochondrial respiratory capacity and elevated ROS levels observed in senescent MIN6 cells (Figure 2f–h). Further validation in primary C57BL/6J mouse islets revealed that natural aging induced excessive basal insulin secretion, compromising glucose‐stimulated insulin secretion, both of which were effectively corrected upon treatment with hAMSC‐sEVs (Figure 2i–k). Mitochondrial metabolic analysis showed that hAMSC‐sEVs reduced basal oxygen consumption rate (OCR) and enhanced glucose‐stimulated OCR and ATP production in aged islets, accompanied by decreased ROS levels (Figure 2l–n). These comprehensive findings reveal that these stem cells attenuate β‐cell senescence by suppressing p16/p53‐p21 signaling and restoring mitochondrial metabolic homeostasis, rescuing insulin secretion in oxidative stress‐induced and naturally aged models.

### 2.3 hAMSC‐sEVs Treat Age‐Related Diabetes in Mice

**FIGURE 3:**
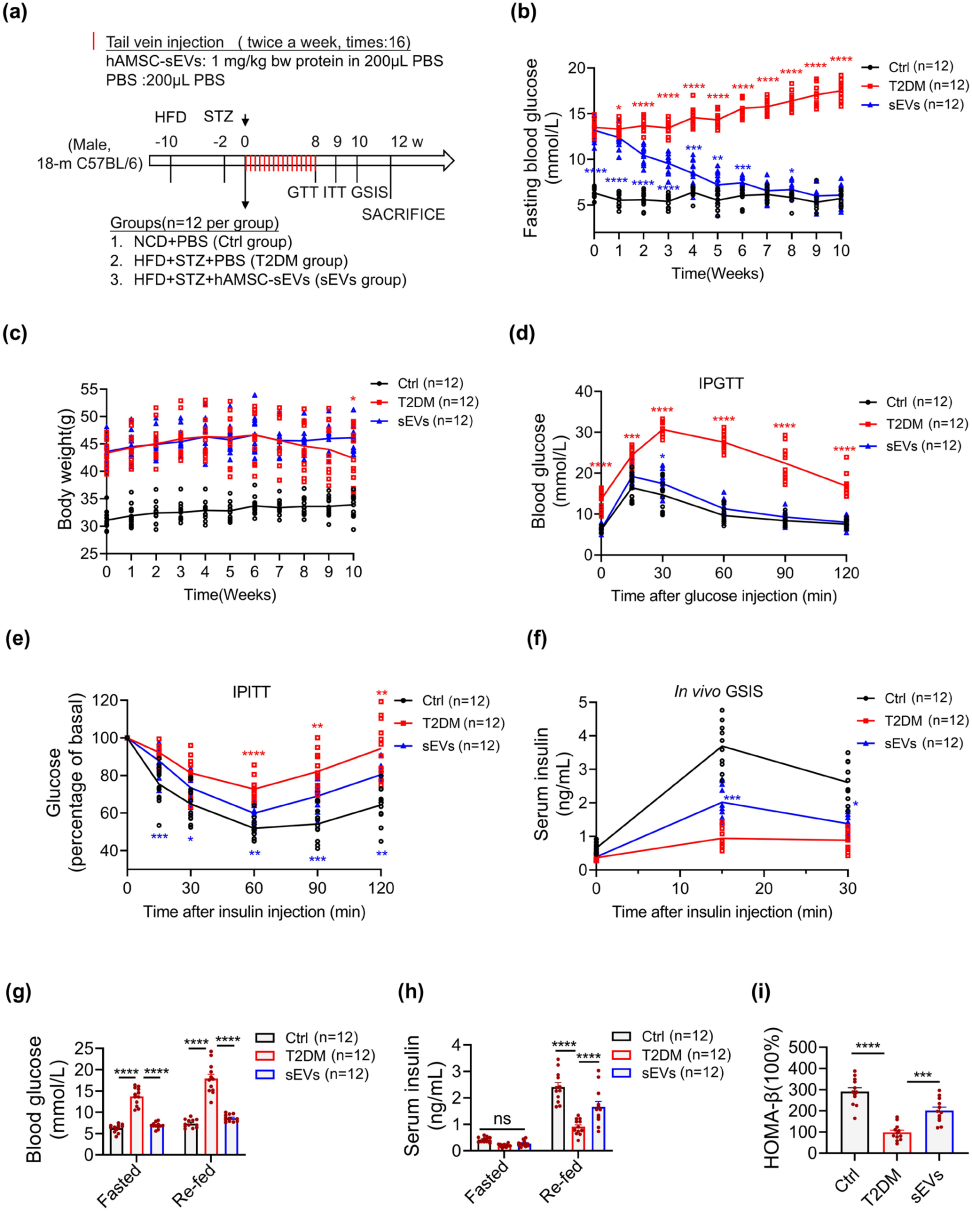
hAMSC‐sEVs treat age‐related diabetes in mice. (a) Schematic of the aged‐diabetic mouse model and hAMSC‐sEV administration. Mice are assigned to three groups: NCD + PBS (Ctrl), HFD + STZ + PBS (T2DM), and HFD + STZ + hAMSC‐sEVs (sEVs). (b) Fasting blood glucose is monitored longitudinally. (c) Body weight is monitored longitudinally. (d) Intraperitoneal glucose tolerance test (IPGTT). (e) Intraperitoneal insulin tolerance test (IPITT). (f) In vivo GSIS is assessed. (g) Blood glucose is measured under fasted/refed states. (h) Serum insulin is measured under fasted/refed states. (i) β‐cell function is assessed by HOMA‐β: HOMA‐β = (FINS [μIU/mL] × FPG [mmol/L]) / (PG2h [mmol/L] + PG1h [mmol/L] − 2 × FPG [mmol/L]). Each dot represents one mouse; lines/bars denote group means (time courses: mean only; bar charts: mean ± SEM). *n* = 12 mice per group. Significance coding: red asterisks, sEVs versus T2DM; blue asterisks, sEVs versus Ctrl. *p < 0.05, **p < 0.01, ***p < 0.001, ****p < 0.0001; ns, not significant.

The demonstrated anti‐senescence effects of hAMSC‐sEVs on β‐cells in vitro motivated our investigation into their therapeutic potential in aged diabetic murine models. Considering the established roles of chronological aging and metabolic stress in β‐cell senescence (Aguayo‐Mazzucato et al. 2017, 2019), we established an aged diabetic mouse model by combining a high‐fat diet with low‐dose streptozotocin (STZ) administration in 18‐month‐old C57BL6/J mice (Figure 3a). This model successfully replicated key diabetic phenotypes, with significant elevations in blood glucose levels and body weight compared with those of normal chow diet (NCD)‐fed controls (Figure 3b,c). PKH26 tracking showed rapid biodistribution with pancreatic enrichment, peaking at 24–48 h and waning by 72–96 h; negligible signals in phosphate‐buffered saline (PBS) controls (Figure S3a–c), with clear co‐localization within insulin^+^ islets at 24 h indicating β‐cell uptake (Figure S3d). Based on our preliminary observations of improvements in glycemic control and insulin secretion (data not shown) mediated by hAMSC, we systematically evaluated the therapeutic efficacy of hAMSC‐sEVs in this model system. Animals were randomized to receive tail‐vein injections of hAMSC‐sEVs or vehicle (PBS); age‐matched NCD‐fed mice injected with PBS served as non‐diabetic controls (Figure 3a). Longitudinal follow‐up revealed a sustained reduction of fasting glycemia over 10 weeks in the sEV group versus T2DM (Figure 3b). Body‐weight trajectories were maintained in the sEV group, whereas untreated T2DM mice displayed a late‐phase decline (≈9% by week 10), resulting in significantly lower weights than the sEV group (Figure 3c). Metabolic characterization using the intraperitoneal glucose tolerance test (IPGTT; week 8) and the intraperitoneal insulin tolerance test (IPITT; week 9) demonstrated blunted glucose excursions and improved insulin responsiveness after hAMSC‐sEV treatment (Figure 3d,e). Given the established role of insulin resistance in exacerbating β‐cell secretory demand and accelerating β‐cell senescence in T2DM (Aguayo‐Mazzucato et al. 2017, 2019), these findings reveal a potential mechanism for hAMSC‐sEV‐mediated protection. To directly assess β‐cell function, we measured serum insulin under fasting and refed conditions and performed an in vivo GSIS assay. hAMSC‐sEV treatment increased insulin secretory capacity (Figure 3f–h), and the homeostasis model assessment of β‐cell function (HOMA‐β) was higher in sEV‐treated mice than in untreated T2DM controls (Figure 3i). Additionally, hAMSC‐sEVs improved peripheral insulin sensitivity, as indicated by enhanced insulin signaling and a metabolic shift toward glucose utilization in liver and white adipose tissue (Figure S4). Together, these results indicate that hAMSC‐sEVs restore β‐cell function and ameliorate hyperglycemia in aged diabetic mice.

### 2.4 hAMSC‐sEVs Alleviate β‐Cell Senescence and Restore Maturation With a Favorable Safety Profile in Aged Diabetic Mice

**FIGURE 4:**
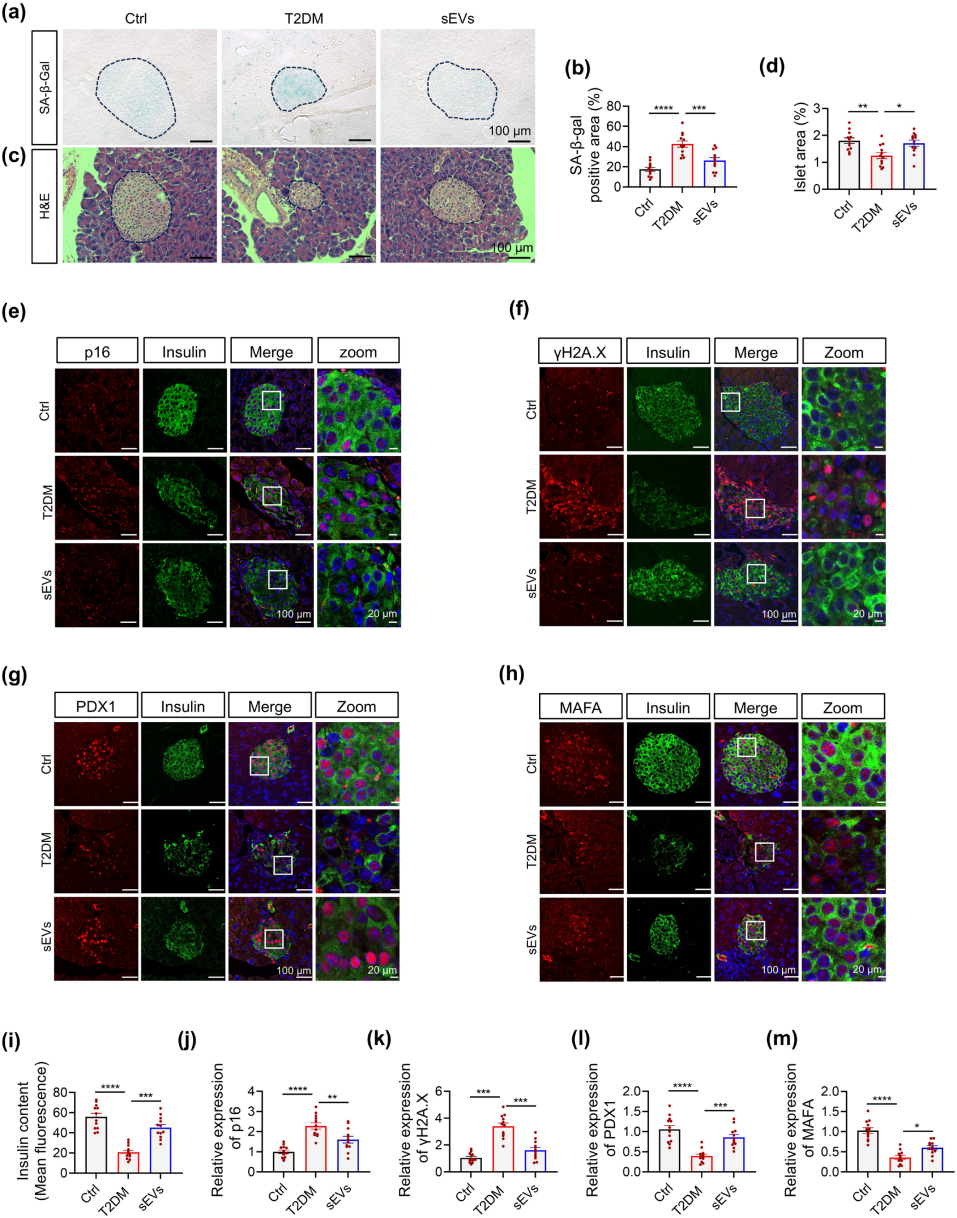
hAMSC‐sEVs alleviate β‐cell senescence and restore maturation in aged diabetic mice. (a) Representative SA‐β‐gal staining of pancreatic islets from Ctrl, T2DM, and sEVs groups. (b) Quantification of SA‐β‐gal–positive area per islet for (a). (c) H&E staining of pancreatic islets. (d) Quantification of islet area in H&E sections for (c). (e) Immunofluorescence (IF) for insulin (green) and p16 (red). (f) IF for insulin (green) and γ‐H2AX (red). (g) IF for insulin (green) and PDX1 (red). (h) IF for insulin (green) and MAFA (red). (i) Quantification of insulin content from IF sections. (j–m) Quantification of IF signals for p16 (j), γ‐H2AX (k), PDX1 (l), and MAFA (m). For panels (a–m), metrics are derived from 15 to 20 islets per mouse across 3–6 non‐adjacent sections; the unit of analysis is the mouse (each dot represents one mouse; group data are presented as mean ± SEM). *n* = 12 per group. Scale bars: 100 μm (overview panels); 20 μm (Zoom). *p < 0.05, **p < 0.01, ***p < 0.001, ****p < 0.0001.

Building on the established link between β‐cell senescence and T2DM progression, we evaluated the therapeutic effects of hAMSC‐sEVs on β‐cell senescence in aged diabetic mice. Significantly reduced senescent islet cells were observed in sEV‐treated mice versus untreated controls upon histochemical analysis with SA‐β‐gal staining (Figure 4a,b). Morphometric analysis showed that sEVs enhanced the islet‐to‐pancreas area ratio in hematoxylin and eosin (H&E)‐stained sections (Figure 4c,d). Immunohistochemical quantification showed marked reductions in senescence markers (p16, γ‐H2AX, insulin‐like growth factor 1 receptor [IGF1R]) in pancreatic islets (Figure 4e,f,j,k and Figure S3e,g). Molecular profiling showed that sEV upregulated β‐cell maturation regulators PDX1, MAFA, and insulin (Figure 4g–i,l,m), aligning with known transcriptional suppression in senescent β‐cells (Aguayo‐Mazzucato 2020). Given the established phenomenon of β‐cell dedifferentiation during senescence (Murao et al. 2022; Song et al. 2022), we examined aldehyde dehydrogenase 1 family member A3 (ALDH1A3) expression, a molecular marker of β‐cell dedifferentiation whose activity dynamically correlates with β‐cell identity and function (Son et al. 2023). sEV administration normalized ALDH1A3 expression (Figure S3f,h), indicating functional preservation.

We systematically analyzed hepatic/renal function, blood lipids, and inflammatory cytokines at 1‐month post‐treatment to evaluate the biosafety of hAMSC‐sEVs. The results showed that the therapeutic intervention did not adversely affect hepatic/renal function or blood lipid profiles in aged diabetic mice (Figure S5a–f). Notably, sEV treatment improved specific hepatic function parameters (alanine aminotransferase [ALT] and triglyceride [TG] levels (Figure S5a,f), while aspartate aminotransferase [AST] and creatinine [CREA] remained comparable across groups (Figure S5b,d)). Significantly elevated blood urea nitrogen [BUN] and total cholesterol [TC] were observed in the disease group (Figure S5c,e). Cytokine profiling revealed that treatment with sEVs reduced SASP factors IL‐1β, IL‐6, and CCL2 (Figure S5g,h,j), but did not significantly impact tissue necrosis factor‐α (TNF‐α) levels (Figure S5i). These data show that hAMSC‐sEVs exert senotherapeutic efficacy, restore β‐cell function, and display a favorable safety profile in aged‐diabetic mouse models.

### 2.5 miR‐21‐5p Mediates hAMSC‐sEV‐Driven β‐Cell Rejuvenation and Metabolic Improvement In Vitro and In Vivo

miRNAs are critical functional components of sEV cargo and contribute substantially to mediating intercellular communication and biological effects (Ragni et al. 2021). We performed comprehensive miRNA sequencing analysis on sEVs isolated from three young hAMSC lines (A10, A13, A15) to identify specific miRNAs responsible for the anti‐senescence properties of hAMSC‐sEVs. The relative abundance of the top 20 miRNAs was quantified based on reads per million mapped reads (Figure S6a,b). Gene ontology (GO) and Kyoto Encyclopedia of Genes and Genomes (KEGG) enrichment analyses of target genes for the six highly expressed miRNAs revealed significant enrichment in senescence‐associated pathways, including DNA damage response, p53 signaling pathway, longevity regulation, autophagy regulation, cell cycle checkpoints, and cytokine signaling networks, among others (Figure S6c,d). These findings show that hAMSC‐sEVs likely exert a multimodal regulation on cellular senescence through coordinated intervention in these interconnected aging‐related networks. After treating senescent MIN6 cells with hAMSC‐sEVs, we observed significant upregulation of four miRNAs: miR‐21‐5p, miR‐100‐5p, miR‐143‐3p, and let‐7i‐5p (Figure S6e). Functional characterization through miRNA‐mimic transfection revealed that miR‐21‐5p overexpression effectively attenuated multiple cellular senescence markers, including SA‐β‐gal activity and the expression of p53, p21, and p16, and concurrently enhanced the expression of β‐cell functional maturation markers such as PDX1 and glucose transporter 2 (GLUT2) (Figure S6f–h). Notably, among the examined miRNAs, only miR‐21‐5p was consistently downregulated in aged β cells (Figure S6i), supporting its role as a key mediator of the anti‐senescence activity of hAMSC‐sEVs. Furthermore, cross‐species analyses revealed an age‐related decline of miR‐21‐5p: in the human islet aging dataset (GSE181066), donor age was inversely correlated with β‐cell miR‐21‐5p (Figure S7a), and miR‐21‐5p was similarly reduced in islets from naturally aged and aged T2DM mice (Figure S7b,c).

**FIGURE 5:**
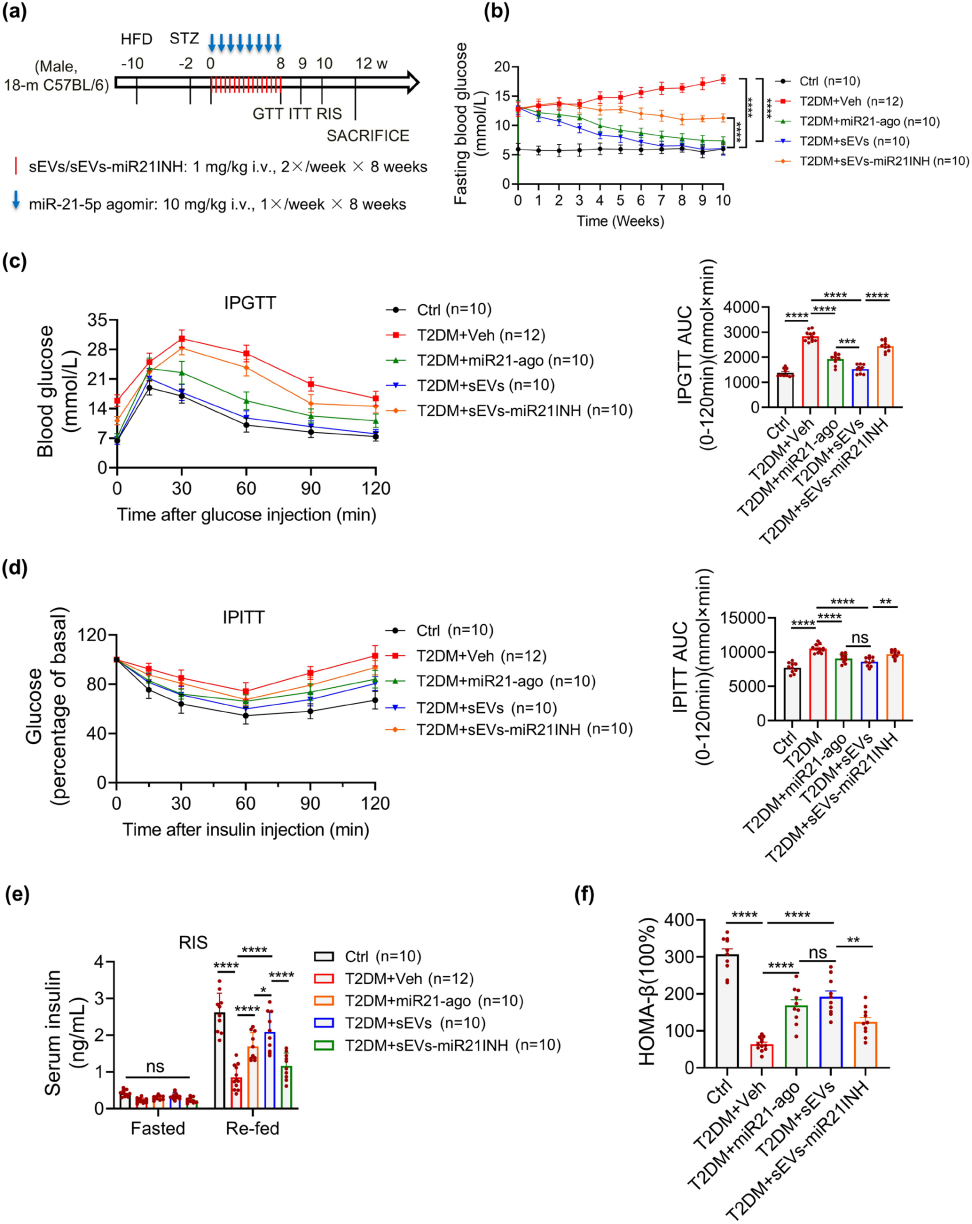
miR‐21‐5p is necessary and partially sufficient for hAMSC‐sEV efficacy in aged diabetic mice. (a) Experimental schema. Eighteen‐month‐old male C57BL/6J mice are rendered diabetic by HFD plus low‐dose STZ, then are treated intravenously for 8 weeks with vehicle, hAMSC‐sEVs (1 mg/kg, twice weekly), miR‐21‐5p agomir (10 mg/kg, once weekly), or miR‐21‐5p‐depleted sEVs (sEVs‐21INH; 1 mg/kg, twice weekly). Groups are: Ctrl (NCD + PBS), T2DM + Veh, T2DM + miR21‐ago, T2DM + sEVs, and T2DM + sEVs‐21INH. sEVs‐21INH are generated by loading an antisense oligonucleotide against miR‐21‐5p with Exo‐Fect and are validated in vitro prior to in vivo use. (b) Fasting blood glucose is monitored over time. (c) IPGTT is performed with AUC analysis. (d) IPITT is performed with AUC analysis. (e) Plasma insulin is measured in fasted versus refed states, capturing refeeding‐induced insulin secretion (RIS). (f) β‐cell function is assessed by HOMA‐β. Each dot represents one mouse; data are presented as mean ± SEM; *n* per panel is indicated in the figure. Two‐tailed tests are used with appropriate multiple‐comparison corrections. *p < 0.05, **p < 0.01, ***p < 0.001, ****p < 0.0001; ns, not significant.

We employed a loss‐of‐function strategy using a miR‐21‐5p inhibitor to establish its functional necessity. Pretreating hAMSC‐sEVs with the inhibitor significantly reduced vesicular miR‐21‐5p (Figure S8a) and blunted their anti‐senescence activity. This was evidenced by a smaller decrease in SA‐β‐gal^+^ cells (Figure S8b,c), incomplete suppression of γ‐H2A.X (Figure S8d,e), attenuated restoration of proliferation in senescent β cells (Figure S8f,g), and diminished rescue of GSIS and insulin synthesis (Figure S8h,i). miR‐21‐5p inhibition also impaired mitochondrial function—with reduced glucose‐stimulated OCR, ATP production, and spare respiratory capacity (Figure S8j,k), and weaker ROS scavenging (Figure S8l). At the molecular level, inhibition attenuated sEV‐mediated downregulation of senescence markers and upregulation of PDX1 at both the mRNA and protein levels (Figure S8m–o). Conversely, miR‐21‐5p overexpression in senescent β cells recapitulated the hAMSC‐sEV phenotype—reducing SA‐β‐gal and γ‐H2AX, restoring proliferation, enhancing GSIS with a modest increase in insulin synthesis, improving mitochondrial respiration and ROS handling, and reinstating PDX1/GLUT2 expression (Figure S9). In vivo, glucose‐insulin phenotyping in aged‐diabetic mice corroborated the necessity and partial sufficiency of miR‐21‐5p for hAMSC‐sEV efficacy (Figure 5). Study schema: Figure 5a. Fasting glycemia improved with sEVs, was partly reproduced by the miR‐21‐5p agomir, and was blunted when vesicular miR‐21‐5p was depleted (Figure 5b). IPGTT curves and AUC showed better glucose tolerance with sEVs, partial benefit with the agomir, and loss of efficacy after inhibition (Figure 5c); IPITT curves and AUC showed parallel effects on insulin sensitivity (Figure 5d). Re‐feeding insulin and HOMA‐β rose with sEVs and more modestly with the agomir, but declined after miR‐21‐5p inhibition (Figure 5e,f). Histology and immunostaining substantiated β‐cell rejuvenation (Figure S10): increased islet area (Figure S10a), reduced SA‐β‐gal^+^ cells (Figure S10b), decreased p16 and restored PDX1 (Figure S10c,d). Together, these data identify miR‐21‐5p as a conserved, β‐cell‐directed effector of hAMSC‐sEVs that drives anti‐senescence remodeling and metabolic improvement, establishing its necessity and partial sufficiency for these benefits in vitro and in vivo.

### 2.6 hAMSC‐sEV‐Derived miR‐21‐5p Suppresses the IL‐6RA/STAT3 Axis to Alleviate β‐Cell Senescence

**FIGURE 6:**
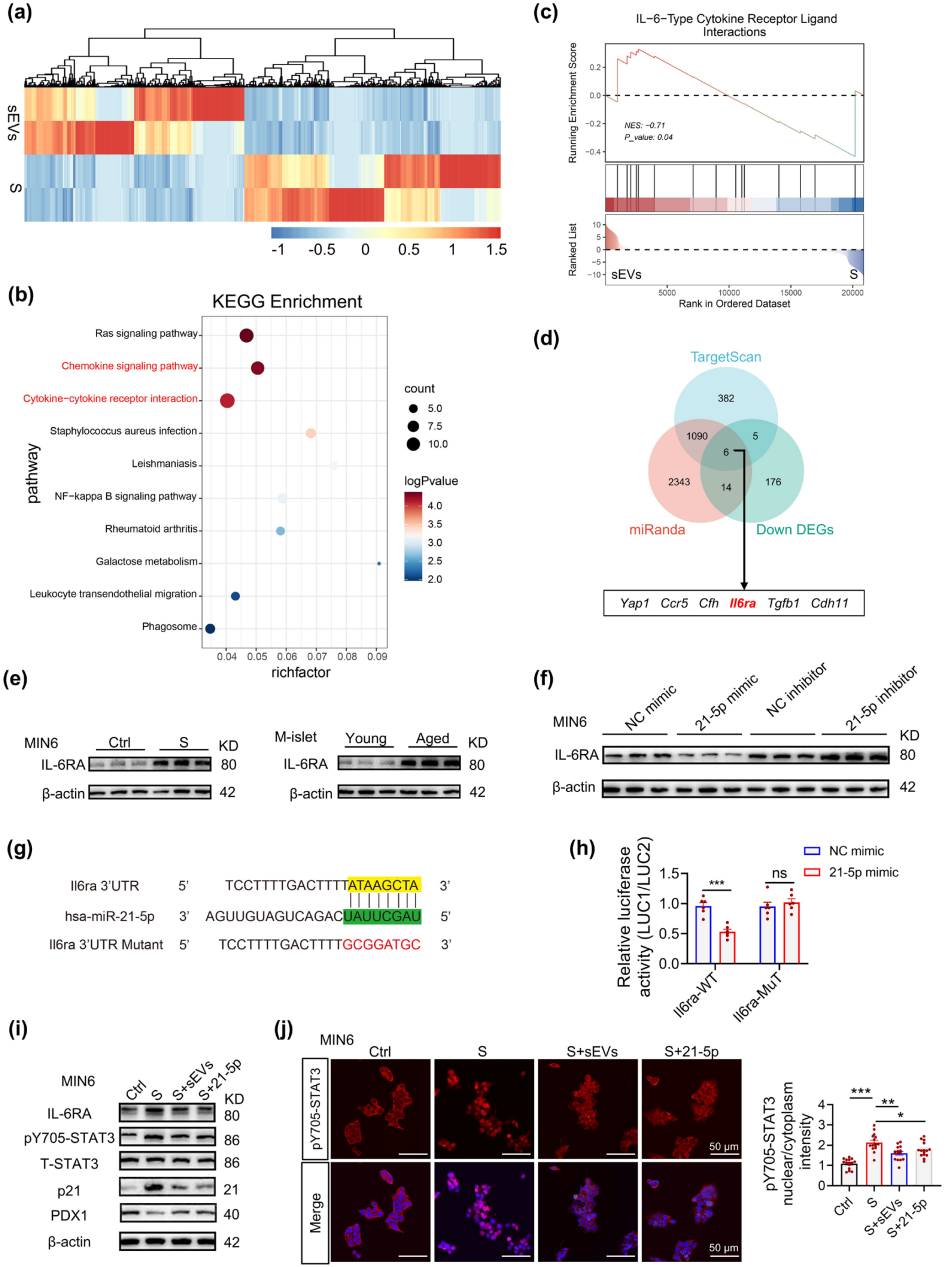
hAMSC‐sEV‐miR‐21‐5p targets the IL‐6RA/STAT3 axis to ameliorate β‐cell senescence. (a) Heatmap shows differentially regulated genes (|log₂FC| > 1, *p* < 0.05) between senescent MIN6 cells (S) and hAMSC‐sEV–treated senescent MIN6 cells (sEVs) by RNA‐seq. (b) KEGG pathway enrichment is performed for genes significantly modulated by hAMSC‐sEVs. (c) Gene set enrichment analysis (GSEA) indicates enrichment for the IL‐6 family cytokine receptor–ligand interaction signature (NES, normalized enrichment score). (d) Venn diagram illustrates the overlap between downregulated DEGs in sEV‐treated senescent MIN6 cells and miR‐21‐5p–predicted targets (TargetScan and miRanda). (e) Western blots show IL‐6RA expression in H₂O₂‐induced senescent MIN6 cells and in islets isolated from young (2‐month) and aged (18‐month) C57BL/6J mice. (f) IL‐6RA protein levels are shown in MIN6 cells transfected with NC mimic, miR‐21‐5p mimic, NC inhibitor, or miR‐21‐5p inhibitor. (g) Schematic shows wild‐type and mutant Il6ra 3′UTR luciferase reporter constructs. (h) Dual‐luciferase assays validate miR‐21‐5p binding to the Il6ra 3′UTR; *n* = 6 per group. (i) Western blots show IL‐6RA, p‐STAT3 (Tyr705), total STAT3, p21, and PDX1 in Ctrl, S, S + sEVs, and S + 21‐5p mimic MIN6 cells. (j) Representative immunofluorescence images (left) and quantification (right) show pY705‐STAT3 nuclear translocation across groups. Scale bar, 50 μm. Each dot represents one field‐of‐view mean (≈30–50 cells), collected across independent experiments; *n* = 12 fields per group from 3 independent experiments. Data are presented as mean ± SEM. *p < 0.05, **p < 0.01, ***p < 0.001; ns, not significant.

We performed mRNA sequencing to analyze transcriptomic changes in senescent MIN6 cells before and after hAMSC‐sEVs treatment to investigate the underlying mechanisms of hAMSC‐sEVs in regulating β‐cell senescence (Figure 6a). KEGG pathway enrichment analysis (*p* < 0.05, |log_2_FC| ≥ 1) revealed that differentially expressed genes were predominantly associated with the cytokine signaling and cytokine–cytokine receptor interaction pathways (Figure 6b). Previous studies have shown significant upregulation of interleukin receptors in senescent β‐cells, with the SASP exhibiting pathway‐specific enrichment within interleukin signaling cascades (Midha et al. 2021). Notably, through the gene set enrichment analysis, the IL‐6‐type cytokine receptor–ligand interactions were identified as the most significantly altered signaling cascade, suggesting that hAMSC‐sEVs might attenuate β‐cell senescence by inhibiting this pathway (Figure 6c).

We hypothesized that hAMSC‐sEVs exert anti‐senescence effects by transferring miR‐21‐5p to senescent MIN6 cells, thereby silencing target genes. To identify key targets, Venn diagram analysis was conducted comparing genes downregulated in hAMSC‐sEVs‐treated senescent cells with miR‐21‐5p targets predicted by two algorithms (TargetScan and miRanda). Six candidate targets (*Yap1, Ccr5*, *Cfh*, *Il6ra*, *Tgfb1*, *Cdh11*) were identified from the analysis (Figure 6d). Among these, IL‐6RA was prioritized because of its established role as a key mediator of IL‐6 signaling, a pathway strongly implicated in cellular senescence across various age‐related pathologies (Fabian et al. 2021; Tyrrell and Goldstein 2021; Forcina et al. 2022; Paldor et al. 2022). Experimental validation revealed consistent IL‐6RA elevation in senescent MIN6 cells and aged murine islets (Figure 6e), complemented by reciprocal expression patterns following miR‐21‐5p modulation: overexpression suppressed while inhibition enhanced IL‐6RA protein levels (Figure 6f). We confirmed a direct regulatory interaction through the 3′‐untranslated region (3′‐UTR) luciferase reporter assays, demonstrating that miR‐21‐5p mediated the *Il6ra* suppression (Figure 6g,h).

Building upon the canonical IL‐6 signaling paradigm mediated through IL‐6R/STAT3 phosphorylation, we systematically investigated the regulatory effects of hAMSC‐sEVs and miR‐21‐5p on this pathway. Mechanistic analyses revealed that hAMSC‐sEVs and miR‐21‐5p effectively suppressed the activation of IL‐6R signaling, as evidenced by diminished tyrosine 705 phosphorylation (pY705‐STAT3) and impaired nuclear shuttling of STAT3 (Figure 6i,j). This pathway inhibition was functionally associated with dual regulatory effects: a marked reduction in senescence‐associated p21expression and a concomitant upregulation of PDX1—a key transcriptional regulator essential for β‐cell functional maintenance (Figure 6i). To further establish causality within the IL‐6RA/STAT3 axis, epistasis assays showed that enforced IL‐6RA expression lacking the 3′‐UTR (thus miR‐21‐5p‐resistant) abrogated the anti‐senescence effects of miR‐21‐5p—re‐increasing SA‐β‐gal positivity, reducing mitochondrial membrane potential (JC‐1 red/green), diminishing GSIS, and restoring IL‐6RA/pY705‐STAT3 and p21 while lowering PDX1—whereas IL‐6RA‐WT did not negate miR‐21‐5p actions (Figure S11a–d). Moreover, forced pathway re‐activation by IL‐6 trans‐signaling (IL‐6‐ts) or constitutively active STAT3 (STAT3‐C) similarly reversed miR‐21‐5p benefits across cellular, mitochondrial, secretory, and molecular readouts (Figure S11e–h). These epistasis results confirm that miR‐21‐5p alleviates β‐cell senescence by targeting the IL‐6RA/STAT3 axis.

In aged diabetic mice, β‐cell IL‐6RA and nuclear pSTAT3 were elevated and were normalized by sEV treatment (Figure S12a,b). In senescent MIN6 cells, Il6ra knockdown reduced SASP/aging transcripts (e.g., Il6ra, Cdkn2a, Trp53, Il1b, Il6; Figure S12c), decreased SA‐β‐gal and γH2AX, restored insulin expression and EdU incorporation (Figure S12d–h), improved GSIS (Figure S12i), and suppressed IL‐6RA/pY705‐STAT3, p21/p16 while upregulating PDX1 (Figure S12j,k). These data independently validate that miR‐21‐5p alleviates β‐cell senescence by targeting the IL‐6RA/STAT3 axis. Collectively, these in vitro and in vivo data identify IL‐6RA as a causal node of β‐cell aging and demonstrate that hAMSC‐sEV‐delivered miR‐21‐5p counteracts senescence by directly silencing IL‐6RA and dampening STAT3 signaling.

### 2.7 MiR‐21‐5p Attenuates β‐Cell Senescence by Suppressing the IL‐6RA/STAT3/MCU Axis to Restore Mitochondrial Calcium‐Redox Coupling

**FIGURE 7:**
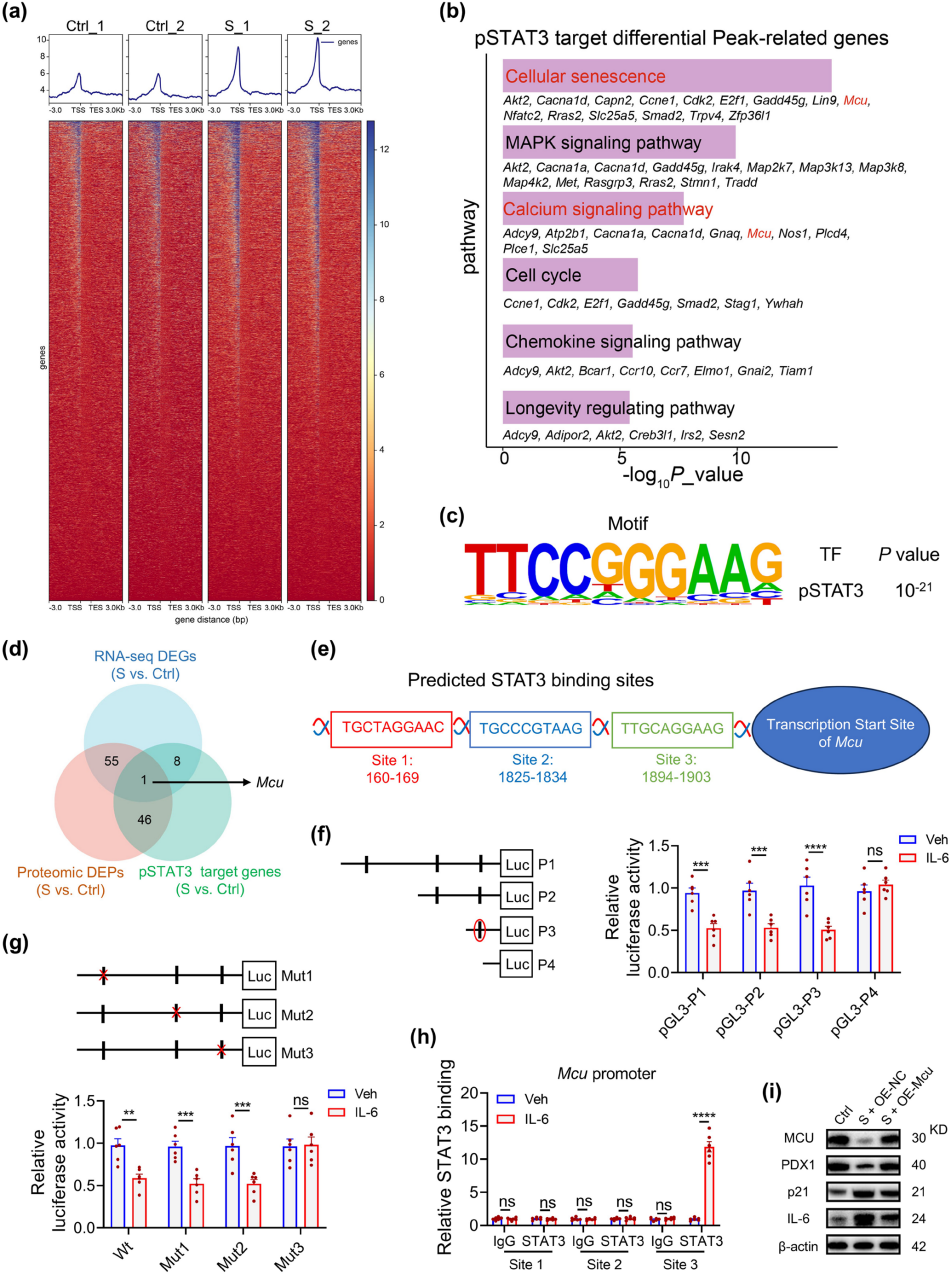
Integrated multi‐omics profiling reveals STAT3‐mediated transcriptional regulation of Mcu in β‐cell senescence. (a) Heatmaps show CUT&Tag‐seq signals of phosphorylated STAT3 (pY705‐STAT3) around transcription start sites (TSSs) in H₂O₂‐induced senescent MIN6 cells (S_1/S_2) versus normal controls (Ctrl_1/Ctrl_2). (b) KEGG pathway enrichment is shown for genes associated with differential pSTAT3 binding peaks; the top six pathways ranked by significance are listed with genes. (c) De novo motif analysis using HOMER identifies a characteristic pSTAT3 motif; motif significance is indicated by grayscale letter height. (d) Venn diagram illustrates convergence of RNA‐seq differentially expressed genes (DEGs, blue), proteomic differentially expressed proteins (DEPs, red), and CUT&Tag binding peaks (green) in senescent (S) versus control (Ctrl) MIN6 cells. (e) JASPAR‐predicted STAT3 binding motifs are mapped in the *Mcu* promoter. (f, g) Luciferase assays assess serial 5′ truncations (f) and site‐directed mutants (g) of the *Mcu* promoter in MIN6 cells after IL‐6 stimulation; *n* = 6 per group. (h) ChIP‐qPCR validates phosphorylation‐dependent STAT3 occupancy at the *Mcu* promoter; *n* = 6 per group. (i) Western blots show MCU, PDX1, p21, and IL‐6 in H₂O₂‐induced senescent MIN6 cells transfected with OE‐*Mcu* or OE‐NC for 48 h. Data are presented as mean ± SEM. **p < 0.01, ***p < 0.001, ****p < 0.0001; ns, not significant.

To delineate STAT3‐mediated transcriptional regulation, we performed Cleavage Under Targets and Tagmentation (CUT&Tag) profiling of Tyr705‐phosphorylated STAT3 (pY705‐STAT3) in senescent (S_1/S_2) and normal MIN6 cells (Ctrl_1/Ctrl_2). Heatmaps showed pSTAT3 peaks centered on transcription start sites (TSSs) with senescence‐associated increases in binding intensity (Figure 7a). Bioinformatic analysis of differentially bound regions indicated enrichment of pSTAT3 targets in cellular senescence, MAPK and calcium signaling, cell‐cycle control, chemokine signaling, and longevity pathways (Figure 7b). De novo motif discovery identified a conserved 10‐bp sequence (*p* = 1.0 × 10^−21^) predictive of pSTAT3 binding (Figure 7c). Integrated RNA‐seq, proteomics, and CUT&Tag analyses converged on the mitochondrial calcium uniporter (MCU) as a pSTAT3‐regulated gene with mechanistic relevance to both senescence and calcium signaling (Figure 7b,d).

We next stimulated MIN6 cells with IL‐6 to probe the direct relationship between pSTAT3 and *Mcu*. IL‐6 induced Y705 STAT3 phosphorylation with reciprocal downregulation of MCU protein by immunoblotting (Figure S13a). Three putative STAT3 motifs (−160/−169, −1825/−1834, and −1894/−1903) were mapped within the Mcu promoter (Figure 7e). IL‐6‐activated pSTAT3 suppressed *Mcu* promoter activity; 5′ deletions and site‐directed mutagenesis localized the critical repressive cis‐element to −1894/−1903 bp (Figure 7f,g). Following IL‐6 treatment, chromatin immunoprecipitation (ChIP) confirmed the enrichment of pSTAT3 at this promoter segment, establishing direct STAT3‐dependent transcriptional repression of MCU (Figure 7h). In islets from aged T2DM mice, miR‐21‐5p was decreased, IL‐6RA and pY705‐STAT3 were elevated, and PDX1/MCU were reduced; hAMSC‐sEVs partially reversed these abnormalities and restored MCU by immunoblotting and immunofluorescence (Figure S14a–d). Consistently, in vivo causality experiments substantiated the signaling mechanism: sEVs and the miR‐21‐5p agomir suppressed IL‐6RA/pY705‐STAT3 and reinstated MCU/PDX1, whereas miR‐21‐5p inhibition abrogated these effects, confirming miR‐21‐5p–IL‐6RA/pSTAT3–MCU axis control in β cells (Figure S10e,f).

**FIGURE 8:**
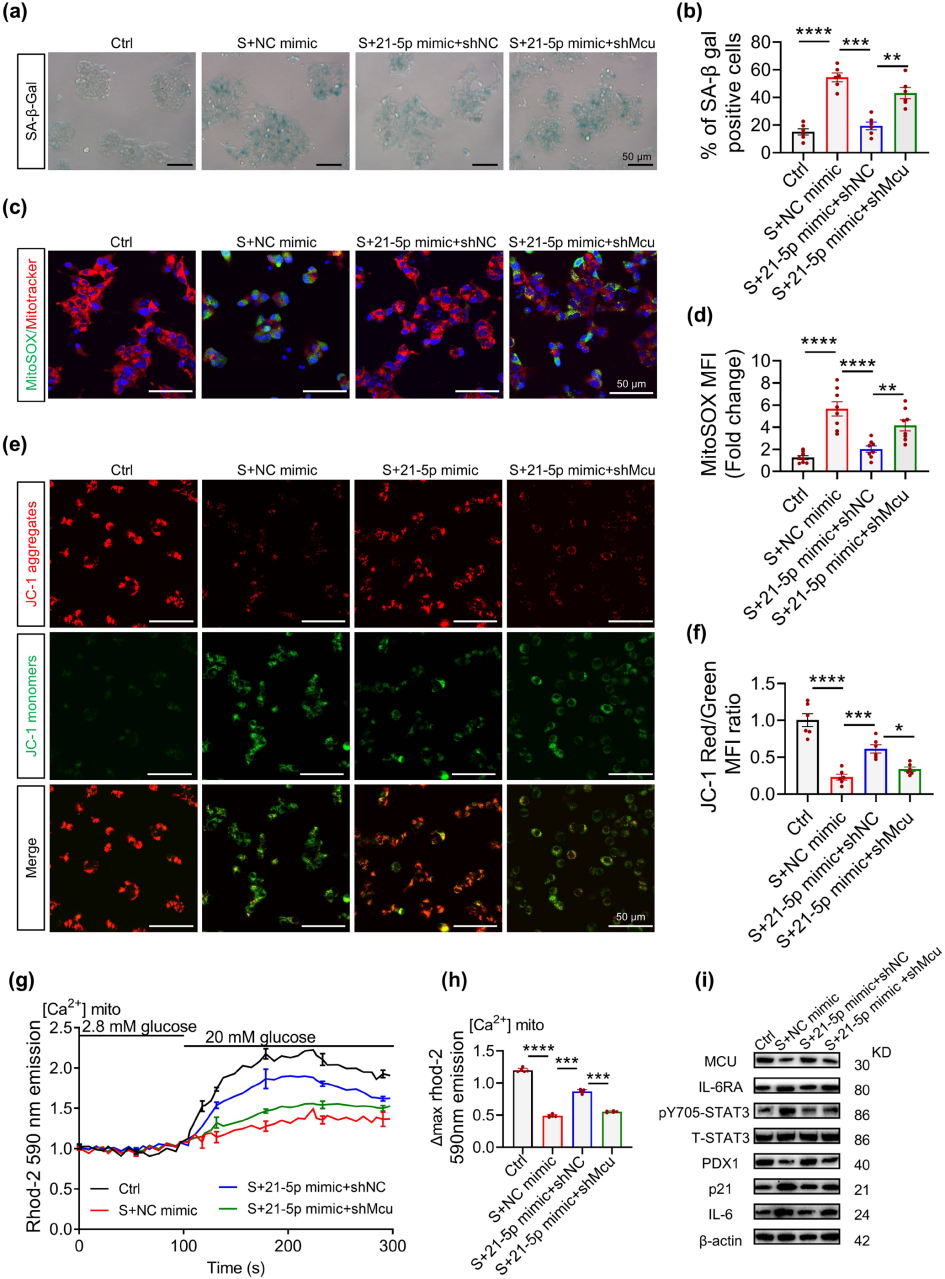
MiR‐21‐5p attenuates β‐cell senescence by suppressing the IL‐6RA/STAT3/MCU axis to restore mitochondrial calcium‐redox coupling. (a–i) H₂O₂‐induced senescence model in MIN6 cells (200 μM, 2 h) with combinatorial interventions of miR‐21‐5p mimic and Mcu‐targeting shRNA (shMcu); cells are analyzed 48 h post‐transfection. (a) Representative SA‐β‐gal staining. (b) Quantification of SA‐β‐gal–positive cells for (a); *n* = 6 per group. Scale bar, 50 μm. (c) Representative co‐staining with MitoSOX (superoxide, green) and MitoTracker (mitochondrial mass, red). Scale bar, 50 μm. (d) Quantification of MitoSOX fluorescence intensity for (c); *n* = 8 per group. (e) Representative JC‐1 staining (red, high ΔΨm aggregates; green, low ΔΨm monomers). Scale bar, 50 μm. (f) Quantification of red/green ratios (ΔΨm index) for (e); *n* = 6 per group. (g, h) Mitochondrial Ca^2+^ levels ([Ca^2+^]ₘᵢₜₒ) are measured with Rhod‐2 after stimulation with 20 mM glucose; (g) shows average fluorescence traces, and (h) shows maximal Rhod‐2 signals (normalized to basal); *n* = 5 per group. (i) Western blots show MCU, IL‐6RA, pY705‐STAT3, total STAT3, p16, p21, and PDX1. Data are presented as mean ± SEM. *p < 0.05, **p < 0.01, ***p < 0.001, ****p < 0.0001.

Recent studies report age‐related declines in mitochondrial Ca^2+^ uptake (Migliavacca et al. 2019; Gherardi et al. 2025) and MCU‐dependent control of senescence via mitochondrial Ca^2+^ signaling (Seegren et al. 2023; Gherardi et al. 2025). In MIN6 β‐cells, MCU was downregulated during senescence; MCU overexpression reduced SA‐β‐gal activity (Figure S13b), lowered p21/IL‐6, and increased PDX1 (Figure 7i). Functionally, senescent cells displayed elevated mitochondrial superoxide (mtSOX) and depolarized membrane potential, both rescued by MCU overexpression (Figure S13c–f). MCU also restored the age‐associated deficit in mitochondrial Ca^2+^ influx, a key determinant of GSIS, thereby rescuing functional output (Figure S13g–i). Rescue experiments in β‐cell senescence models further revealed that miR‐21‐5p‐mediated improvements in SA‐β‐gal activity (Figure 8a,b), mtSOX reduction (Figure 8c,d), mitochondrial membrane potential (ΔΨm) restoration (Figure 8e,f), and calcium uptake enhancement (Figure 8g,h) were substantially attenuated upon MCU knockdown. Through molecular analyses, MCU knockdown compromised miR‐21‐5p‐induced downregulation of senescence markers (p21/IL‐6) and upregulation of the β‐cell maturation marker PDX1 (Figure 8i). Collectively, these findings reveal that hAMSC‐sEVs mitigate mitochondrial dysfunction and reverse β‐cell senescence via miR‐21‐5p‐mediated suppression of the IL6RA/STAT3/MCU signaling axis.

## 3 Discussion

In summary, we establish a mechanistically grounded, cell‐free strategy that rejuvenates pancreatic β cells and improves glycemic control in aged diabetes, thereby clarifying the physiological and therapeutic relevance of our findings.

Aging is an important risk factor for T2DM, which is characterized by β‐cell senescence leading to dysfunction (Tuduri et al. 2022). Targeting β‐cell senescence may potentially help reverse diabetes progression. sEVs from embryonic/young mesenchymal stem cells reduce senescence markers (Dorronsoro et al. 2021; Sanz‐Ros et al. 2022, 2025; Yu et al. 2023; Bi et al. 2025), making them potential therapies for β‐cell senescence‐induced dysfunction. Our findings revealed that hAMSC‐sEVs effectively counteracted β‐cell aging by reversing senescence, maintaining β‐cell identity, and restoring insulin secretion. Functionally, this translated into improved fasting glycemia, glucose tolerance, in vivo GSIS, and insulin sensitivity in aged diabetic mice, indicating disease‐modifying potential rather than transient glucose lowering. Notably, concordant benefits were observed across three complementary models (oxidative stress–induced β‐cell senescence in vitro, naturally aged islets ex vivo, and aged T2DM in vivo), strengthening physiological generalizability. This finding provides a novel anti‐aging strategy for treating age‐related diabetes by targeting senescent pancreatic β cells.

To investigate how hAMSC‐sEVs regulate senescent β‐cells, we employed oxidative stress‐induced senescent β‐cells in vitro and in naturally aged mouse islets in vivo, co‐incubating them with sEVs. Multidimensional assessments, including analysis of senescence markers (SA‐β‐gal activity, γ‐H2AX, EDU, p16 staining), islet function (GSIS, islet perfusion), and mitochondrial metrics (OCR, ROS), showed that hAMSC‐sEVs rescued β‐cell function by suppressing senescence biomarkers (p53/p21/p16) and restoring mitochondrial metabolic homeostasis. Consistent with reports in liver, kidney, spleen, muscle, and skin, stem‐cell sEVs suppress p53/p21 activity (Yu et al. 2023; Bi et al. 2025) and recalibrate mitochondrial homeostasis and oxidative phosphorylation; our β‐cell data extend this cross‐tissue pattern (Shuler et al. 2020; Dutra et al. 2021). In vivo trials showed that immediate intravenous sEV administration in newly diagnosed aged diabetic mice significantly enhanced islet function and insulin sensitivity. Reduced senescent β‐cell burden and restored β‐cell identity biomarkers, such as PDX1 and MAFA, explained the movement of islets, while the effects of sEV on peripheral insulin‐sensitive organs (liver, adipose, muscle) possibly contributed to glycemic control. Sun et al. reported that mesenchymal stem cell‐derived sEVs reversed peripheral insulin resistance by restoring insulin receptor substrate 1 (IRS1) and protein kinase B (AKT) phosphorylation (Sun et al. 2018), supporting this mechanism. Additionally, sEVs improved liver function (ALT reduction) and lipid profiles (TG levels), reflecting their pleiotropic effects. Notably, intravenously administered sEVs predominantly accumulated in the liver, pancreas, kidney, and adipose tissues (Su et al. 2025), suggesting that the observed systemic metabolic improvements represent coordinated actions from various organs. Importantly, no hepatorenal toxicity signals were observed in aged diabetic mice receiving hAMSC‐sEVs, supporting translational feasibility alongside efficacy and on‐target engagement within the pancreas. Beyond these conserved anti‐aging features, our work identifies a β‐cell–specific physiological axis restored by hAMSC‐sEVs: mitochondrial Ca^2+^ uptake–bioenergetic/redox coupling–GSIS. When investigating the molecular components within sEVs, we detected miRNAs in three lines of young hAMSC‐sEVs and identified miR‐21‐5p, miR‐100‐5p, miR‐221‐3p, miR‐143‐3p, miR‐127‐3p, and let‐7i‐5p as the six most enriched miRNAs. Through miRNA mimic‐based anti‐aging screening assays in vitro, we revealed that miR‐21‐5p served as the critical anti‐senescence factor in hAMSC‐sEVs, mediating functional recovery of senescent β‐cells. Inhibiting it in sEVs neutralized their anti‐senescence effects. To strengthen causality, we performed in vivo gain‐ and loss‐of‐function studies—miR‐21‐5p agomir and miR‐21‐5p–depleted sEVs—which demonstrated that miR‐21‐5p is necessary and partially sufficient for the therapeutic effect. These experiments define a causal pathway (miR‐21‐5p → IL‐6RA/STAT3 → MCU) that ties β‐cell rejuvenation directly to GSIS physiology, moving beyond correlative markers to organ‐specific function.

miR‐21‐5p, a highly conserved miRNA, is closely associated with metabolic disease development, including obesity and diabetes (Saliminejad et al. 2019; Lhamyani et al. 2021). Deficiency of miR‐21 impairs GSIS in pancreatic β‐cells and induces glucose intolerance (Liu et al. 2022). Hypoxia significantly promotes β‐cell apoptosis, and miR‐21‐mimics markedly enhance β‐cell viability under hypoxic conditions (Chen et al. 2020). However, the expression profile and regulatory role of miR‐21 in senescent β‐cells remain unknown. Our results showed that miR‐21‐5p was downregulated in senescent β‐cells, and hAMSC‐sEVs restored β‐cell function by replenishing miR‐21‐5p. This anti‐senescence activity of miR‐21‐5p has also been reported in myeloid stem cells (Qi et al. 2024), endothelial progenitor cells (Cao et al. 2024), CD4^+^ T cells (Xiong et al. 2021), and oocytes (Yang et al. 2020). However, conflicting findings from studies identify miR‐21‐5p as a biomarker of endothelial senescence (Zhang et al. 2017), where sEVs from senescent endothelial cells deliver miR‐21‐5p to promote senescence via DNA methylation and replication regulation (Mensa et al. 2020). These seemingly contradictory findings may be caused by differences in cell types, senescence stages, and disease models. Accordingly, we do not claim β‐cell exclusivity for miR‐21‐5p. Its therapeutic predominance here reflects context and target availability: senescent β‐cells upregulate IL‐6RA—a direct miR‐21‐5p target—and uniquely couple MCU‐dependent mitochondrial Ca^2+^ uptake to GSIS, thereby amplifying the functional benefit of miR‐21‐5p delivery.

Recent studies show that IL‐6 signaling activation is strongly associated with cellular senescence, and IL‐6 signaling blockade improves senescent phenotypes and delays progression of multiple age‐related diseases (Xu, Guo, et al. 2015; Xu, Tchkonia, et al. 2015; Paldor et al. 2022; Hoffman et al. 2023). In previous studies, significantly elevated plasma IL‐6 levels were confirmed in patients with T2DM (Kado et al. 1999), with high circulating IL‐6 serving as an independent predictor of T2DM (Spranger et al. 2003). IL‐6RA is the critical molecule for IL‐6‐mediated JAK/STAT3 signal transduction (Xu, Guo, et al. 2015; Xu, Tchkonia, et al. 2015). Through transcriptome sequencing/analysis, target prediction software, and luciferase reporter assays, we identified IL‐6RA as a direct target of miR‐21‐5p. IL‐6RA was significantly upregulated in oxidative stress‐induced senescent β‐cells, naturally aged mouse islets, and β‐cells from aged T2DM mice. miR‐21‐5p derived from hAMSC‐sEV reversed β‐cell senescence by targeting IL‐6RA/STAT3 signaling activation. An integrated analysis of RNA‐seq, proteomics, and CUT&Tag sequencing data from senescent vs. non‐senescent β‐cells revealed MCU as a transcriptional target of pSTAT3, mechanistically linked to its dual regulatory roles in cellular senescence and calcium signaling. Mitochondrial calcium (mtCa^2+^) uptake through MCU combines calcium homeostasis with energy metabolism (Baughman et al. 2011). In β‐cells, MCU maintains normal GSIS function by tightly regulating mitochondrial Ca^2+^ concentration (Allen and Tessem 2022). Emerging evidence shows that age‐dependent decline is implicated in mitochondrial Ca^2+^ uptake capacity (Migliavacca et al. 2019; Gherardi et al. 2025). Furthermore, enhancing MCU‐dependent calcium uptake improves cellular senescence by increasing mitochondrial energetics (Gherardi et al. 2025). Moreover, mitochondrial dysfunction induced by reduced mitochondrial Ca^2+^ uptake has been identified as central to inflammatory aging (Seegren et al. 2023). In this study, we confirmed through reporter assays and ChIP‐quantitative polymerase chain reaction that MCU expression was transcriptionally suppressed by pSTAT3, revealing a novel regulatory mechanism underlying diminished mitochondrial Ca^2+^ uptake in senescent β‐cells. Restoring MCU expression (via overexpression or miR‐21‐5p delivery) significantly improved mitochondrial function, reversed cellular senescence markers, and enhanced insulin secretion. Together, these data position miR‐21‐5p as a molecular “release valve” that relieves an IL‐6RA/STAT3‐mediated brake on MCU, thereby re‐establishing β‐cell‐specific Ca^2+^–bioenergetic coupling central to GSIS and metabolic health.

Although our study centers on miR‐21‐5p, hAMSC‐sEVs are multimolecular carriers; proteins, lipids, and diverse RNAs may act synergistically to rejuvenate β cells. Prior evidence shows exosomal protein cargo (e.g., VEGF) enhances islet viability and insulin secretion (Keshtkar et al. 2020), adipocyte‐derived EV proteins augment GSIS (Lopez‐Otin et al. 2023), lipid constituents regulate vesicle uptake/signaling (Kitada et al. 2014; Mathieu et al. 2019), and circRNAs influence β‐cell identity and insulin secretion (Xu, Guo, et al. 2015; Xu, Tchkonia, et al. 2015; Liu et al. 2023). While miR‐21‐5p emerges as a principal effector here, contributions from non‐miRNA cargos cannot be excluded; future studies will employ enzymatic digestion and cargo fractionation/deconvolution to quantify their relative effects.

In summary, we establish a cell‐free, anti‐β‐cell senescence strategy in which hAMSC‐sEVs deliver miR‐21‐5p to suppress the IL‐6RA/STAT3/MCU axis, rescuing mitochondrial Ca^2+^‐redox coupling, rejuvenating β cells, and restoring GSIS and glycemic control in aged‐diabetic mice. These findings bridge molecular mechanisms to organ‐level benefits and position sEV‐based delivery as a promising anti‐aging therapeutic modality for age‐related diabetes.

## 4 Materials and Methods

### 4.1 Cell Culture

hAMSCs were procured from the State Key Laboratory of Reproductive Medicine (First Affiliated Hospital of Nanjing Medical University) under ethical approval (2012‐SR‐128) with donor‐informed consent, isolated using established protocols (Qin et al. 2022), and cultured in α‐MEM (Cat#12571‐063, Gibco) supplemented with 5% human platelet lysate (UltraGRO‐hPL, Cat#HPCPLCRL50, Helios Bioscience), 1% L‐glutamine (Cat#25030, Gibco), and 0.03% heparin (Cat#H32022088, Changzhou Qianhong Bio‐pharma Co. Ltd.) (P3‐5 passages), while MIN6 cells (provided by Xiao Han, Nanjing Medical University) were maintained in DMEM (Cat#11965092, Gibco) containing 15% FBS (Cat#A5670701, Gibco), 10 mM HEPES (Cat#15630130, Gibco), and 50 μM β‐mercaptoethanol (Cat#M3148, Sigma‐Aldrich) (Miyazaki et al. 1990), with primary murine islets cultured in RPMI‐1640 (Cat#A1049101, Gibco)/10% FBS following standard isolation protocols (Nie et al. 2013), all under standard culture conditions (37°C, 95% air/5% CO₂).

### 4.2 Characterization of hAMSC and sEVs

hAMSCs were immunophenotypically characterized using the Human MSC Analysis Kit (Cat#562245, BD Biosciences) through dual‐labeling flow cytometry: positive markers (CD73/CD90/CD105, 1 μg/mL, 4°C/30 min) and hematopoietic lineage exclusion markers (cocktail: CD11b/CD19/CD34/CD45/HLA‐DR) with isotype‐matched controls, performed on 80%–90% confluent cells dissociated into single‐cell suspensions using FACS buffer (PBS supplemented with 10% FBS and 0.1% sodium azide). Tri‐lineage differentiation capacity was validated through osteogenic/adipogenic/chondrogenic induction per established protocols (Liu, Huang, et al. 2021; Liu, Jiang, et al. 2021), with lineage‐specific matrix deposition visualized by histochemical staining (Nikon Eclipse Ni‐U).

sEVs were isolated from P3–5 hAMSC‐conditioned serum‐free α‐MEM (Cat#05‐201‐1U, Bioind) via sequential centrifugation (300 *g*/10 min → 2000 *g*/30 min → 12,000 *g*/30 min) followed by 0.22 μm filtration and dual ultracentrifugations (120,000 *g*/3 h at 4°C), with purified vesicles characterized through TEM (Hitachi, HT‐7700), nanoparticle tracking analysis (NanoFCM N30E) for size distribution, and immunoblotting verification of exosomal markers (CD9/CD63/TSG101) with calnexin exclusion, alongside miRNA quantification using TRIzol‐extracted RNA (Cat#15596026, Invitrogen), all performed according to established methodologies (Liu, Huang, et al. 2021; Liu, Jiang, et al. 2021; Dixson et al. 2023).

### 4.3 Establishment of Aged Diabetic Mouse Model and Treatments

All experimental procedures were conducted in accordance with the National Institutes of Health Guidelines for Laboratory Animals and approved by Nanjing Medical University's Animal Care Committee (IACUC‐NJMU1702023). Eighteen‐month‐old male C57BL6/J mice from Nanjing Medical University's Animal Center were randomly assigned to either a normal chow diet (NCD) or a high‐fat diet (HFD, Cat#D12492, Research Diets) group for 8 weeks. HFD‐fed mice subsequently received three consecutive daily tail vein injections of 45 mg/kg streptozotocin (STZ, 1% in citrate buffer pH 4.5) (Cat#60256ES80, Yeasen, Shanghai, China) after a 12‐h fast. Successfully modeled mice with random blood glucose > 11.1 mmol/L were randomly assigned to either the hAMSC‐sEVs treatment group (sEVs group) or T2DM control group (T2DM group), while age‐matched NCD‐fed mice served as the healthy control group (Ctrl group) (*n* = 6 per group). All animals were maintained under standard specific pathogen‐free conditions (24°C, 45%–55% humidity, 12‐h light/dark cycle) with ad libitum access to food/water throughout the study. The sEVs group received a dose of hAMSC‐sEVs (1 mg/kg body weight), administered via tail vein injection twice a week for a duration of 8 weeks. Concurrently, both the Ctrl group and T2DM group were administered 200 μL of PBS via tail vein injections. sEV protein quantification was performed using bicinchoninic acid (BCA) assay (Cat#P0010, Beyotime, Shanghai, China) per the manufacturer's protocol. All aliquots were thawed overnight at 4°C and processed within 24 h post‐thawing.

### 4.4 Cell Model Preparation and Administration

MIN6 cells at 70% confluency were exposed to hydrogen peroxide (H_2_O_2_) (0–300 μM gradient concentration range, Cat#88597, Sigma‐Aldrich) for 2 h, followed by PBS washing and continued culture in exosome‐depleted complete medium for 48 h to establish premature senescence, with senescence‐associated phenotypic analyses identifying 200 μM as the optimal concentration. Subsequent sEV treatment (0–100 ng/mL) was initiated post‐hydrogen peroxide exposure in sEV‐supplemented complete medium for 48 h, including parallel PBS‐washed controls and fresh medium blanks for experimental standardization (Yu et al. 2021).

### 4.5 hAMSC‐sEVs Cellular Internalization and Biodistribution in Mice

sEVs were fluorescently labeled using PKH26 (Cat#MINI26‐1KT, Sigma‐Aldrich) per the manufacturer's protocol, where 200 μg sEVs were incubated with 1 mL PKH26 solution (RT, 5 min, dark), followed by staining termination with FBS and ultracentrifugation (120,000 *g*, 70 min, 4°C). Purified PKH26‐sEVs were resuspended in PBS and co‐cultured with senescent MIN6 cells (37°C, 24 h) prior to cytoskeletal staining with Phalloidin (Cat#ab176753, 1:1000, Abcam) and nuclear counterstaining with Hoechst 33342 (Cat#C1028, Beyotime, Shanghai, China) for fluorescence microscopy. For in vivo tracking, aged diabetic mice received PKH26‐sEVs (1 mg/kg body weight in 200 μL PBS) via tail vein injection, with PBS‐injected controls. Biodistribution was analyzed 24 h post‐injection using IVIS Lumina LT III (PerkinElmer, λex/em = 551/567 nm). Pancreata were fixed in 4% PFA for confocal microscopy (Olympus FV1200) with insulin/Hoechst co‐staining to verify pancreatic sEV localization.

### 4.6 Metabolic Characters Analysis

Glucose metabolism was assessed through fasting blood glucose (FBG, 14‐16 h fast) and intraperitoneal challenges: glucose tolerance test (IPGTT, 1 g/kg), insulin tolerance test (IPITT, 1 U/kg, 4‐6 h fast), and glucose‐stimulated insulin secretion (GSIS, 3 g/kg, 12 h fast) (Yap et al. 2022), with tail vein blood glucose quantified by Glucometer Elite (Abbott, Oxon, UK) and serum insulin levels measured via ELISA (Cat#MS100, EZ assay, Shenzhen, China). Systemic inflammation markers (IL‐1β/IL‐6/TNF‐α/CCL2) were analyzed using RayBiotech Inflammation Array (Cat#AAM‐INF‐1, RayBiotech, Peachtree Corners, GA, USA), while hepatic/renal functions and blood lipids were evaluated through biochemical profiling (Hitachi, 7100).

### 4.7 Senescence‐Associated β‐Galactosidase (SA‐β‐Gal) Staining

Cellular senescence was assessed using SA‐β‐gal staining (Cat#C0602, Beyotime, Shanghai, China) through PBS‐washed cell fixation (15 min, RT) followed by overnight chromogenic incubation (37°C) with subsequent thorough PBS washing and microscopic quantification, while tissue sections were processed through cryosectioning prior to parallel staining protocols.

### 4.8 Immunofluorescence Staining

Immunofluorescence analysis was performed on 5‐μm paraffin‐embedded pancreatic sections using primary antibodies (Table S1) with 4°C overnight incubation, followed by PBST (PBS containing 1% Tween 20) washing and 37°C 1‐h secondary antibody incubation, counterstained with Hoechst 33342 prior to confocal imaging (Olympus, FV1200), with similar protocols applied to cultured cells.

### 4.9 GSIS and Islet Perfusion Assay

Following 48 h treatments (mimic/shRNA transfection/sEVs), MIN6 cells and murine islets underwent 30‐min glucose‐free KRBH (Krebs‐Ringer bicarbonate‐HEPES) preincubation, followed by 1 h glucose challenge (cells: 2.0/20 mM; islets: 3.3/16.7 mM) with subsequent acid‐ethanol insulin extraction. Islet perfusion assays employed size‐matched islets (*n* = 200/group) from aged C57BL/6J mice (18‐month) cultured in exosome‐depleted RPMI 1640 (100 ng/μL sEVs vs. vehicle, 48 h) followed by KRBH equilibration (0 mM glucose, 37°C/overnight), with dynamic glucose stimulation (0 → 20 mM) executed through sequential perfusion phases: initial low‐glucose priming (125 μL/min × 20 min), reduced‐flow collection phase (< 1 mL/min × 5 min, 1‐min interval sampling), and sustained high‐glucose perfusion (15 min), with perfusate insulin quantified via DNA‐normalized radioimmunoassay.

### 4.10 Oxygen Consumption Rate (OCR) Measurements

Cellular bioenergetic profiling was performed using the Seahorse XF24 Analyzer (Agilent Technologies, MA, USA) to quantify OCR in treated pancreatic islets (40–60 islets/well) or MIN6 β‐cells (5 × 10^4^ cells/well), preconditioned for 4 h in assay medium (0.2% BSA/2 mM glucose) followed by sequential injections of mitochondrial modulators: 20 mM glucose, oligomycin (5 μM islets/4 μM MIN6), 4 μM FCCP, and rotenone/antimycin A (5 μM islets/1 μM MIN6), enabling protein‐normalized quantification of basal respiration, glucose‐stimulated respiration, ATP‐linked production, and maximal respiratory capacity.

### 4.11 Mitochondrial Superoxide and Intracellular ROS Detection

Mitochondrial superoxide levels were quantified through sequential staining with 1 μM MitoSOX Green (Cat#M36006, Thermo Fisher, USA) and 100 nM MitoTracker Red (Cat#M7512, Thermo Fisher, USA) under light‐protected 37°C incubation (30 min each), followed by triplicate PBS washing and nuclear counterstaining with Hoechst 33342, while intracellular ROS levels were assessed via 10 μM DCFH‐DA (Cat# S0033M, Beyotime, Shanghai, China) loading (37°C/20 min) in MIN6 cells/dispersed islets, with all samples subjected to triplicate PBS washing and confocal microscopy imaging (Olympus FV1200) using standardized emission filters (MitoSOX: 488/510 nm; MitoTracker: 579/599 nm; DCF: 488/525 nm).

### 4.12 Mitochondrial Membrane Potential (ΔΨm) Assessment

Mitochondrial membrane potential (ΔΨm) in treated MIN6 cells was assessed using the JC‐1 assay kit (Cat#HY‐K0601, MedChemExpress, USA) per manufacturer's protocol, with 2 μM JC‐1 staining (37°C, 15–20 min) followed by dual PBS washes and confocal imaging (Olympus FV1200) to quantify aggregate/monomer fluorescence ratios, where reduced J‐aggregate (red: Ex/Em 585/590 nm) to monomer (green: Ex/Em 510/527 nm) ratios indicated mitochondrial depolarization.

### 4.13 Mitochondrial Ca^2+^ Dynamics Assessment

Mitochondrial Ca^2+^ dynamics in MIN6 cells (5 × 10^4^ cells/cm^2^, poly‐L‐lysine‐coated dishes) were assessed through Rhod‐2 AM‐based confocal imaging following 24 h adhesion and 48–72 h post‐intervention incubation (transfection/sEVs), with cells pre‐equilibrated in 2.8 mM glucose KRBH (37°C/5% CO₂, 1.5 h) before 1X Rhod‐2 AM (Cat#S1062M, Beyotime, Shanghai, China) loading (37°C/30 min) and triplicate KRBH washing. Real‐time calcium dynamics were captured using an Olympus FV1200 confocal microscope equipped with a 37°C/5% CO₂ chamber, with baseline fluorescence (F₀) recorded for 2 min in 2.8 mM glucose KRBH followed by acute stimulation with 20 mM glucose. Time‐lapse images were acquired at 5‐s intervals over a 5‐min period using a 63× oil immersion objective (Ex/Em: 552/581 nm), with fluorescence intensities quantified via Image J software and normalized as Δ*F*/*F*₀ (*F* = instantaneous fluorescence value, *F*₀ = baseline value).

### 4.14 Quantitative Real‐Time Polymerase Chain Reaction (qPCR)

Total RNA was extracted utilizing TRIzol reagent (Cat#15596026, Invitrogen, USA). Subsequently, the concentrations of RNA were assessed by optical density measurements. Reverse transcription of mRNA (Cat#R333, Vazyme, China) and miRNA (Cat#MR201, Vazyme) was performed according to Vazyme's instructions. Real‐time PCR amplification was carried out in triplicate. ChamQ SYBR qPCR Master Mix (Cat#Q341‐02, Vazyme) was used to create cDNA fragments for use in quantitative PCR. β‐actin and U6 were used to normalize the levels of mRNAs and miRNAs, respectively. Relative mRNA expression was assessed using the 2^−ΔΔ*Ct*^ method. The primer sequences used for qPCR are listed in Table S2.

### 4.15 Western Blot Analysis

Cell, islet, and sEV samples were lysed in RIPA buffer (Cat#89900, Thermo, USA). The protein concentration was determined by BCA assay (Cat#P0010, Beyotime, China). Equal amounts of protein (20ug) were separated by SDS‐PAGE and subsequently transferred to a PVDF membrane (Cat#IPVH00010, Millipore, USA). The membrane was blocked for 1 h at RT and then incubated with primary antibodies overnight at 4°C. The following day, the membrane was incubated with enzyme‐labeled secondary antibodies for 1 h at RT and exposed to ECL exposure solution (Cat#34577, Thermo, USA). Finally, visualization of proteins was obtained using a Bio‐Rad imaging system (Bio‐Rad, Hercules, CA, USA). The primary and secondary antibodies utilized were listed in Table S1. The immunostaining intensity of protein blots was measured using ImageJ software.

### 4.16 sEV Transfection

An miR‐21‐5p inhibitor and negative control miRNA inhibitor (RiboBio, Guangzhou, China) was transfected into hAMSC‐sEVs by using an Exo‐Fect siRNA/miRNA Transfection Kit (Cat#EXFT10A‐1, System Biosciences, USA) as described in the manufacturer's guidelines.

### 4.17 RNA Interference and Gene Overexpression

Short hairpin RNAs (shRNAs) targeting Il6ra or Mcu, along with MCU overexpression plasmids (OE‐MCU) and negative control plasmid (OE‐NC), were synthesized by Beijing Qingke Biotechnology Co. Ltd. shRNA sequences are in Table S2. MIN6 cells at 50%–70% confluence in 6‐well plates were transfected with shRNAs or plasmids using Lipofectamine 3000 (Cat#L3000015, Invitrogen, USA) per the manufacturer's protocol. Knockdown/overexpression efficacy was confirmed 48 h post‐transfection before cell harvest for downstream analysis.

### 4.18 Dual‐Luciferase Reporter Assay

The wild‐type or mutated Il6ra 3′‐UTR fragments (predicted miR‐21‐5p binding sites) were cloned into pmirGLO vectors (Tsingke, Beijing, China), and HEK293T cells were co‐transfected with 40 nM miR‐21‐5p mimics/NC mimics and 120 ng recombinant plasmids using Lipofectamine 3000. Luciferase activity was measured 48 h post‐transfection with a Dual‐Luciferase Assay Kit (Cat#E1910, Promega, USA), with firefly luciferase signals normalized to Renilla luciferase internal controls.

### 4.19 Chromatin Immunoprecipitation Assay (ChIP)

Chromatin immunoprecipitation was performed in MIN6 cells cross‐linked with 1% formaldehyde (37°C, 10 min). Cells were washed with PBS, lysed in 200 μL buffer, and sonicated to shear chromatin (200–500 bp fragments). Lysates were immunoprecipitated with anti‐p‐STAT3 (Tyr705) antibody (Cat#9145, 1:100, CST) or IgG control using protein A/G magnetic beads (Cat#88803, Thermo, USA) and herring sperm DNA. DNA‐protein complexes were eluted (1% SDS/1.1 M NaHCO3, 65°C, 6 h) and purified via PCR purification kit (Vazyme). ChIP‐qPCR primers are listed in Table S2.

### 4.20 MiRNA‐Sequencing and RNA‐Sequencing Analysis

For miRNA characterization in hAMSC‐sEVs, small RNA libraries were generated from TRIzol‐extracted RNA (three biological replicates: A10/A13/A15) using the TruSeq Small RNA Library Prep Kit (Illumina, San Diego, CA, USA), followed by single‐end 50 bp sequencing on the Illumina HiSeq 2500 (LC‐Bio Technology CO. Ltd., Hangzhou, China) with cluster generation via the cBot v2 system. RNA integrity was verified by the NanoDrop ND‐1000 (A260/280 > 1.8; A260/230 > 2.0) and Bioanalyzer 2100 (RIN > 8.0).

For senescence‐associated transcriptome profiling, TRIzol‐isolated RNA from three experimental groups—H_2_O_2_‐induced senescent MIN6 cells (senescent model, S), senescent sEV‐treated group (sEVs), and untreated controls (Ctrl)—was processed through NEBNext Ultra II directional library construction. PE150 sequencing on Illumina NovaSeq 6000 (LC‐Bio Technology CO. Ltd., Hangzhou, China) yielded 40 million reads/sample, enabling DESeq2‐based analysis (v1.34.0) of sEV‐mediated transcriptional regulation.

### 4.21 Cleavage Under Targets and Tagmentation (CUT&Tag) Sequencing

CUT&Tag sequencing was performed on MIN6 cells pretreated with Concanavalin A‐coated magnetic beads and permeabilized with digitonin (0.01%), followed by chromatin immunotargeting using anti‐p‐STAT3 (Tyr705, Cat#9145, CST) under stringent wash conditions (50 mM HEPES, 300 mM NaCl). Adapter‐ligated genomic fragments were amplified via 12‐cycle PCR (KAPA HiFi HotStart) and subjected to PE150 sequencing (LC‐Bio Technology Co. Ltd., Hangzhou, China) at 20 million reads/sample for STAT3‐binding motif analysis using HOMER (v4.11) with default parameters (*p* < 1e‐5).

### 4.22 Statistical Analysis

Data were analyzed by two‐tailed Student's *t*‐tests (pairwise), one‐way ANOVA with Tukey's post hoc (multi‐group), or two‐way ANOVA with Benjamini‐Hochberg correction (metabolic assays). Results are expressed as a mean ± SEM, and a *p* < 0.05 was considered to be statistically significant. All analyses were executed in GraphPad Prism v8.0.1.

## Author Contributions

**Lei Xiao:** conceptualization, methodology, investigation, formal analysis, data curation, writing – original draft, writing – review and editing. **Zicheng Zhang:** methodology, validation, visualization, writing – review and editing. **Tong Li:** software, visualization, validation. **Yuyin Jiang:** methodology. **Yuanxin Liu:** methodology. **Tingting Lv:** methodology. **Lianju Qin:** conceptualization, resources, writing – review and editing. **Yunxia Zhu:** conceptualization, resources, funding acquisition, writing – review and editing, supervision. **Wei Tang:** conceptualization, funding acquisition, project administration, supervision, writing – review and editing.

## Funding

This work was supported by the National Natural Science Foundation of China, 62231013, 81770773, 82470840, 82270844; Jiangsu Province Key R&D Plan Social Development, BE2023774; Research Incubation Startup Fund of Jiangsu Province Geriatric Hospital, FHQD202302.

## Conflicts of Interest

The authors declare no conflicts of interest.

## Supporting information


**Appendix S1:** Supporting Information.

**Table S1:** The list of antibodies.

**Table S2:** The list of primer sequence, shRNA sequence.

## Data Availability

The data that support the findings of this study are available on request from the corresponding author. The data are not publicly available due to privacy or ethical restrictions.
