# Supplementary material for: Small Extracellular Vesicles From Human Amniotic Membrane Mesenchymal Stem Cells Rejuvenate Senescent β Cells and Cure Age‐Related Diabetes in Mice
Source: Aging Cell. 2025 Dec 14;25(1):e70327. doi: 10.1111/acel.70327 (PMC12741205; doi:10.1111/acel.70327)
Supplement: Supplementary file 1 — Appendix S1: Supporting Information. [file ACEL-25-e70327-s003.pdf]

Small extracellular vesicles from human amniotic membrane mesenchymal stem cells rejuvenate senescent β cells and cure age-related diabetes in mice

Lei Xiao[1], Zicheng Zhang[1], Tong Li[1], Yuyin Jiang[1], Yuanxin Liu[1], Tingting Lv[2], Lianju Qin[3,*], Yunxia Zhu[2,*], Wei Tang[1,*]

[1]Department of Endocrinology, Geriatric Hospital of Nanjing Medical University, Nanjing, Jiangsu, 210024, China.

[2]Key Laboratory of Human Functional Genomics of Jiangsu Province, Department of Biochemistry and Molecular Biology, Nanjing Medical University, Nanjing, Jiangsu, 211166, China.

[3]State Key Laboratory of Reproductive Medicine, Center of Clinical Reproductive Medicine, First Affiliated Hospital, Nanjing Medical University, Nanjing 210029, China.

[*]Corresponding

Wei Tang, Department of Endocrinology, Geriatric Hospital of Nanjing Medical University, Nanjing, Jiangsu, 210024, China.
E-mail addresses: drtangwei@njmu.edu.cn
ORCID: https://orcid.org/0000-0003-0466-4587

Yunxia Zhu, Key Laboratory of Human Functional Genomics of Jiangsu Province, Department of Biochemistry and Molecular Biology, Nanjing Medical University, Nanjing, Jiangsu, 211166, China.
E-mail addresses: zhuyx@njmu.edu.cn
ORCID: https://orcid.org/0000-0002-4597-4445

Lianju Qin, State Key Laboratory of Reproductive Medicine, Center of Clinical Reproductive Medicine, First Affiliated Hospital, Nanjing Medical University, Nanjing 210029, China.
E-mail addresses: ljqin@njmu.edu.cn

The PDF file includes:
Supplementary Figures S1 to S14.

Table of contents:

Supplementary figures:

Supplementary Figure S1. Characterization of hAMSC and sEVs derived from hAMSCs.

Supplementary Figure S2. Oxidative stress-induced β-cell senescence model in MIN6 cells.

Supplementary Figure S3. hAMSC-sEVs accumulate in pancreas and are taken up by β-cells, while normalizing IGF1R and ALDH1A3 in aged diabetic islets.

Supplementary Figure S4. hAMSC-sEVs enhance insulin signaling and metabolic reprogramming in peripheral insulin-target tissues.

Supplementary Figure S5. Systemic metabolic and inflammatory profiling post-hAMSC-sEV intervention.

Supplementary Figure S6. miRNA cargo profiling and functional validation in hAMSC-sEVs.

Supplementary Figure S7. Cross-species reduction of miR-21-5p in aging human β cells and mouse islets.

Supplementary Figure S8. miR-21-5p mediates hAMSC-sEV-driven senescent β-cell rejuvenation.

Supplementary Figure S9. miR-21-5p mimics the role of hAMSC-sEVs in anti-β -cell senescence.

Supplementary Figure S10. miR-21-5p underlies hAMSC-sEV-mediated β-cell rejuvenation in aged diabetic mice.

Supplementary Figure S11. miR-21-5p alleviates β-cell senescence by targeting the IL-6RA/STAT3 axis.

Supplementary Figure S12. Alleviating β-cell senescence via IL-6RA: hAMSC-sEVs downregulate IL-6RA and knockdown rejuvenates β-cells.

Supplementary Figure S13. Mcu overexpression exacerbates H_2O_2-induced mitochondrial dysfunction and cellular senescence in MIN6 cells.

Supplementary Figure S14. hAMSC-sEVs restore the islet miR-21-5p/IL-6RA/STAT3/MCU axis in aged diabetic mice.

Supplementary figures and legends

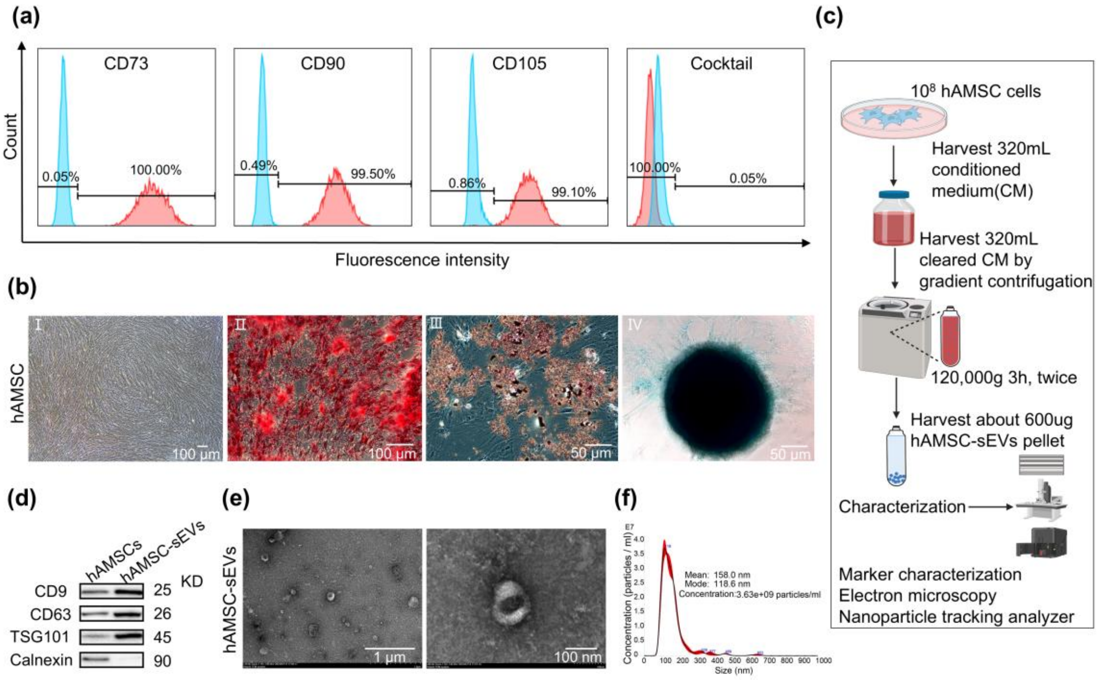

Supplementary Figure S1. Characterization of hAMSC and sEVs derived from hAMSCs. (a) Flow cytometry confirms hAMSC identity: >98% are positive for MSC markers (CD73, CD90, CD105) and <2% are positive for hematopoietic markers (CD11b, CD19, CD34, CD45, HLA-DR). **(b)** hAMSCs display fibroblast-like morphology (I) and differentiate along three lineages into osteoblasts (II), adipocytes (III), and chondrocytes (IV). Scale bars: 100 µm (I-II); 50 µm (III-IV). **(c)** Flowchart illustrates the hAMSC-sEV isolation workflow and quality-control steps. **(d)** Western blots show EV markers (CD9, CD63, TSG101) in sEVs and absence of the cellular protein calnexin. **(e)** TEM reveals cup-shaped vesicles. Scale bars: 1 µm (left); 100 nm (right). **(f)** NTA indicates a predominant particle size distribution of 80-200 nm.

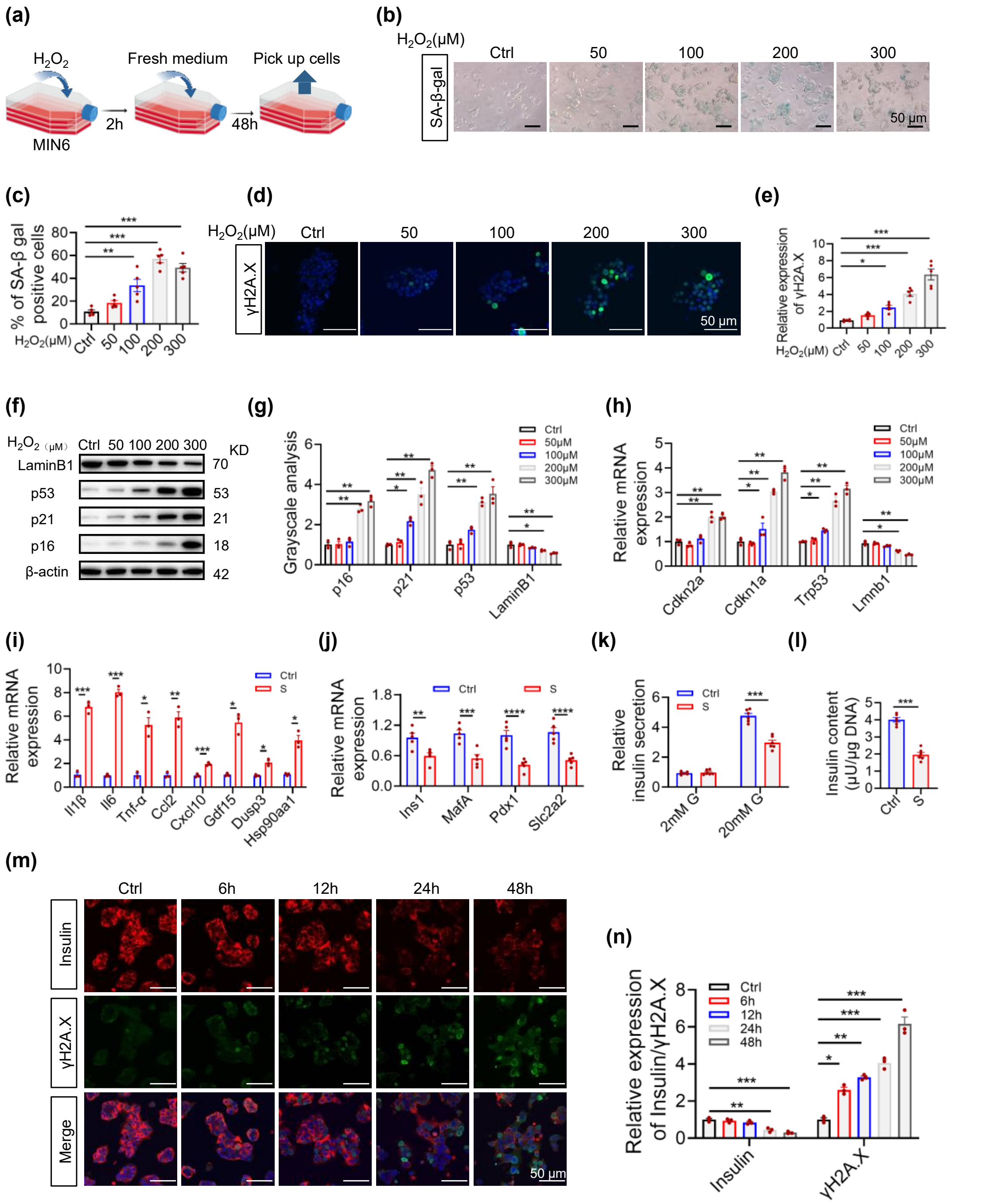

Supplementary Figure S2. Oxidative stress-induced β-cell senescence model in MIN6 cells. (a-h) Senescence is induced by H_2O_2 treatment (50, 100, 200, or 300 μM) or vehicle (Ctrl) for 2 h, followed by 48 h in standard medium. **(a)** Experimental timeline. **(b)** SA-β-gal staining. Scale bars, 50 μm. **(c)** Quantification of SA-β-gal$^+$ cells; n = 5 per group. **(d)** γ-H2AX immunofluorescence. Scale bars, 50 μm. **(e)** γ-H2AX intensity; n = 5 per group. **(f)** Western blots of senescence markers (p53, p21, p16, Lamin B1). **(g)** Densitometry for (f); n = 3 per group. **(h)** Relative mRNA levels of senescence genes (*Cdkn2a, Cdkn1a, Trp53, Lmnb1*); n = 3 per group. **(i-l)** β-cell senescence (S) is induced with H_2O_2 (200 μM, 2 h) followed by 48 h in standard medium. **(i)** Relative mRNA levels of SASP genes (*Il1b, Il6, Tnf, Ccl2, Cxcl10, Gdf15, Dusp3, Hsp90aa1*); n = 3 per group. **(j)** Relative mRNA levels of β-cell maturation

markers (Ins1, MafA, Pdx1, Slc2a2); n = 5 per group. **(k)** GSIS; n = 6 per group. **(l)** Intracellular insulin content; n = 6 per group. **(m-n)** MIN6 cells treated with H_2O_2 (200 μM, 2 h) are collected for staining at 6, 12, 24, and 48 h. **(m)** Co-staining for γ-H2AX (red) and insulin (green). Scale bars, 50 μm. **(n)** Quantification of γ-H2AX and insulin intensities; n = 3 per group. Each dot represents one independent replicate; data are presented as mean ± SEM. **P* < 0.05, ***P* < 0.01, ****P* < 0.001.

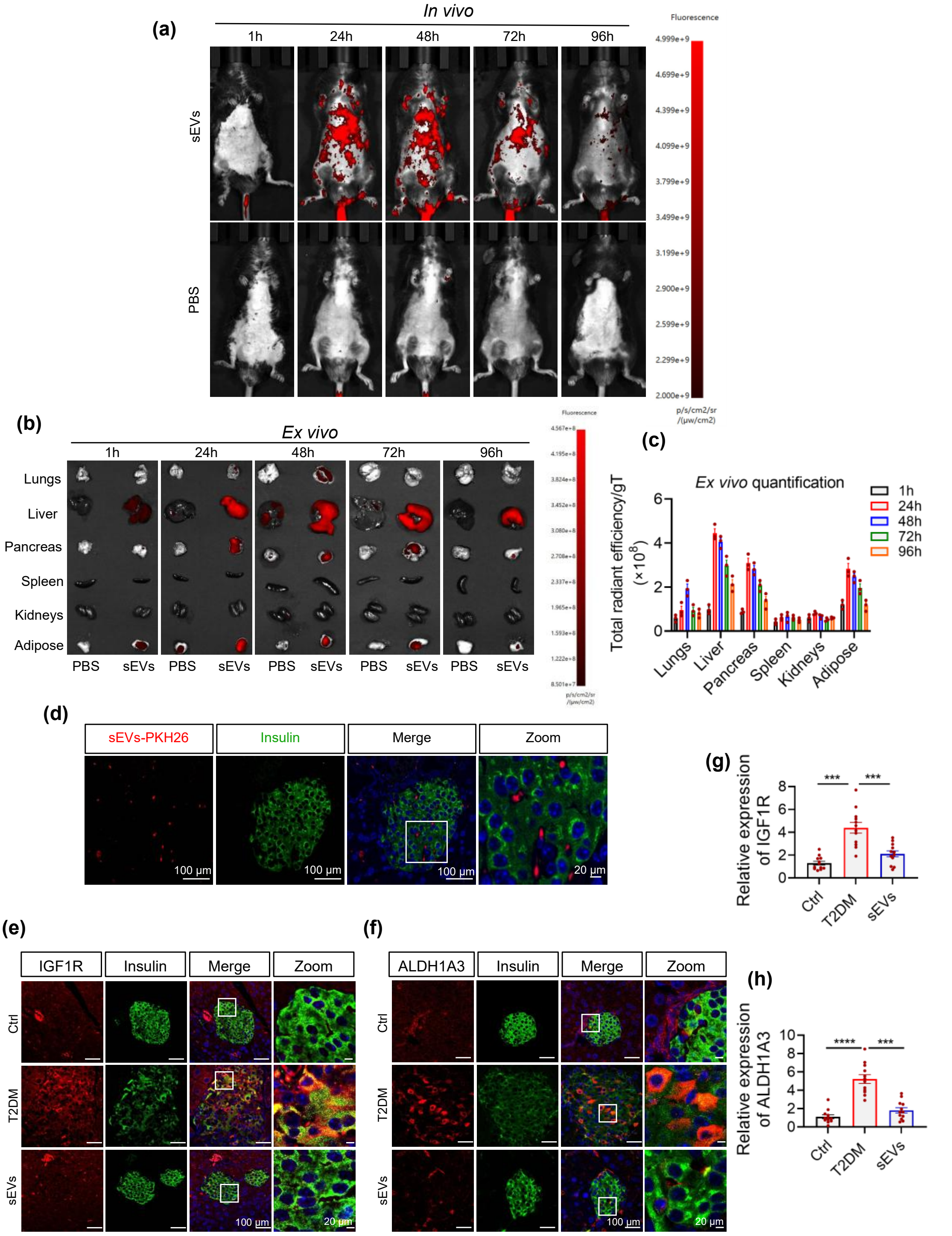

Supplementary Figure S3. hAMSC-sEVs accumulate in pancreas and are taken up by β-cells, while normalizing IGF1R and ALDH1A3 in aged diabetic islets. (a) In vivo imaging. Representative whole-body fluorescence at 1, 24, 48, 72, and 96 h after intravenous PKH26-labeled hAMSC-sEVs or PBS in aged T2DM mice. **(b)** Ex vivo imaging. Fluorescence of dissected organs (lungs, liver, pancreas, spleen, kidneys, adipose) at the indicated times. **(c)** Quantification. Organ signals are expressed as total radiant efficiency per gram tissue $[p/s]/[\mu W/cm2]/gT$[p/s]/[μW/cm2]/gT; fixed-size ROIs are used and background

from time-matched PBS controls is subtracted (IVIS, Living Image v4.7.2). **(d)** Islet uptake. Immunofluorescence for insulin (green) in islets from mice receiving PKH26-labeled sEVs (red) shows β-cell uptake. **(e)** IGF1R. Insulin (green) and IGF1R (red) IF in islets from Ctrl, T2DM, and sEVs groups. **(f)** ALDH1A3. Insulin (green) and ALDH1A3 (red) IF in islets from the same groups. **(g-h)** Quantification. IGF1R **(g)** and ALDH1A3 **(h)** IF intensities per islet; $n = 12$ mice per group. Each dot represents one mouse (mean of 15-20 islets across 3-6 non-adjacent sections); data are presented as mean ± SEM. ***$P < 0.001$, ****$P < 0.0001$. Scale bars: 100 μm (overview panels); 20 μm (Zoom).

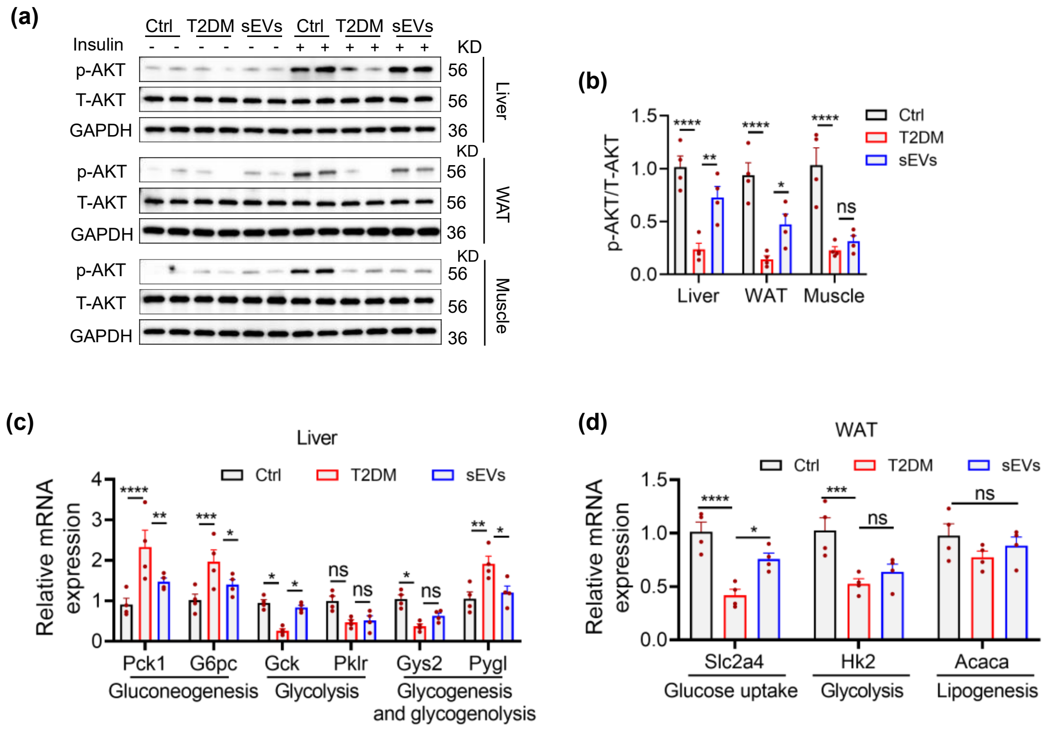

Supplementary Figure S4. hAMSC-sEVs enhance insulin signaling and metabolic reprogramming in peripheral insulin-target tissues. (a) Western blots show p-AKT, total AKT (T-AKT), and GAPDH in liver, white adipose tissue (WAT), and skeletal muscle from Ctrl, T2DM, and sEV-treated mice with or without insulin stimulation. **(b)** Quantification shows p-AKT/T-AKT ratios across Ctrl, T2DM, and sEVs groups. **(c)** Hepatic expression is measured for key metabolic genes: gluconeogenesis (*Pck1, G6pc*), glycolysis (*Gck, Pklr*), and glycogen metabolism (*Gys2, Pygl*). **(d)** WAT expression is measured for glucose uptake (*Slc2a4/GLUT4*), glycolysis (*Hk2*), and lipogenesis/de novo lipogenesis (*Acaca/ACC1*). Each dot represents one independent experiment; data are presented as mean ± SEM. **P* < 0.05, ***P* < 0.01, ****P* < 0.001, *****P* < 0.0001; ns, not significant.

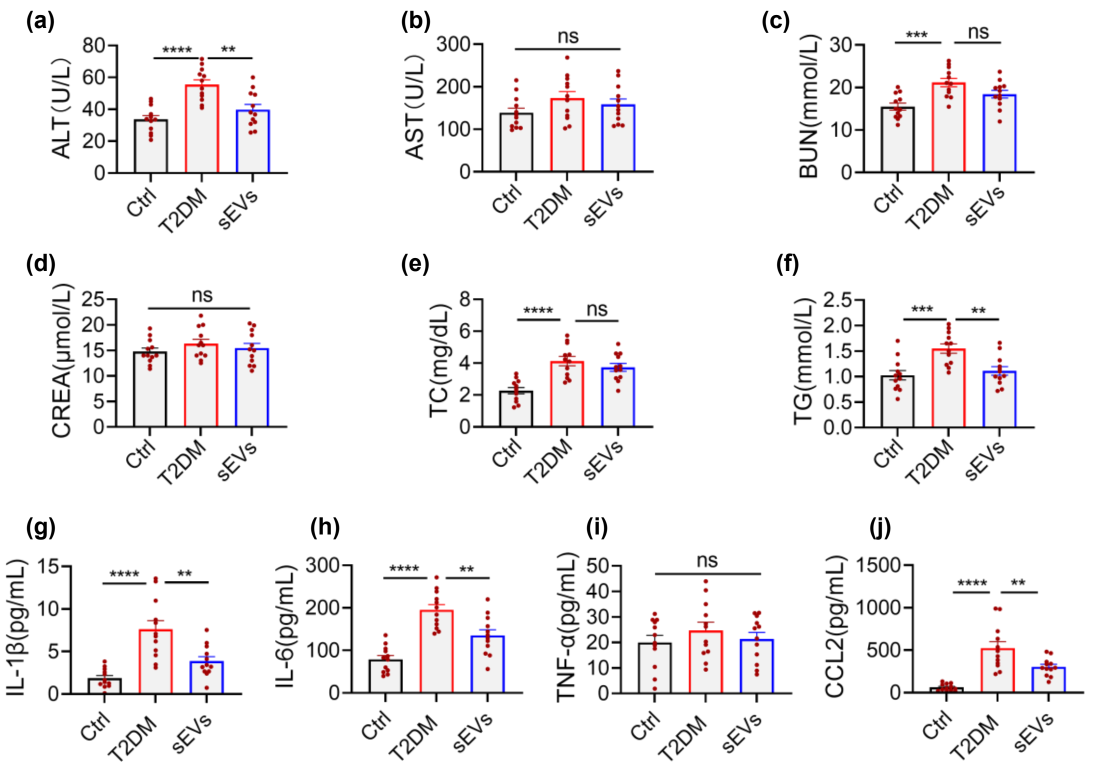

Supplementary Figure S5. Systemic metabolic and inflammatory profiling post-hAMSC-sEV intervention.

(a–j) Metabolic parameters and inflammatory cytokines are quantified 4 weeks after the final hAMSC-sEV infusion in Ctrl, T2DM, and sEVs groups. Mice are fasted overnight, and blood is collected from the retro-orbital plexus; serum is isolated for assays. **(a)** Alanine aminotransferase (ALT). **(b)** Aspartate aminotransferase (AST). **(c)** Blood urea nitrogen (BUN). **(d)** Creatinine (CREA). **(e)** Total cholesterol (TC). **(f)** Triglycerides (TG). **(g)** Interleukin-1β (IL-1β). **(h)** Interleukin-6 (IL-6). **(i)** Tumor necrosis factor-α (TNF-α). **(j)** C-C motif chemokine ligand 2 (CCL2). Data are presented as mean ± SEM; n = 12 mice per group. ns, not significant; $^{**}P < 0.01$, $^{***}P < 0.001$, $^{****}P < 0.0001$.

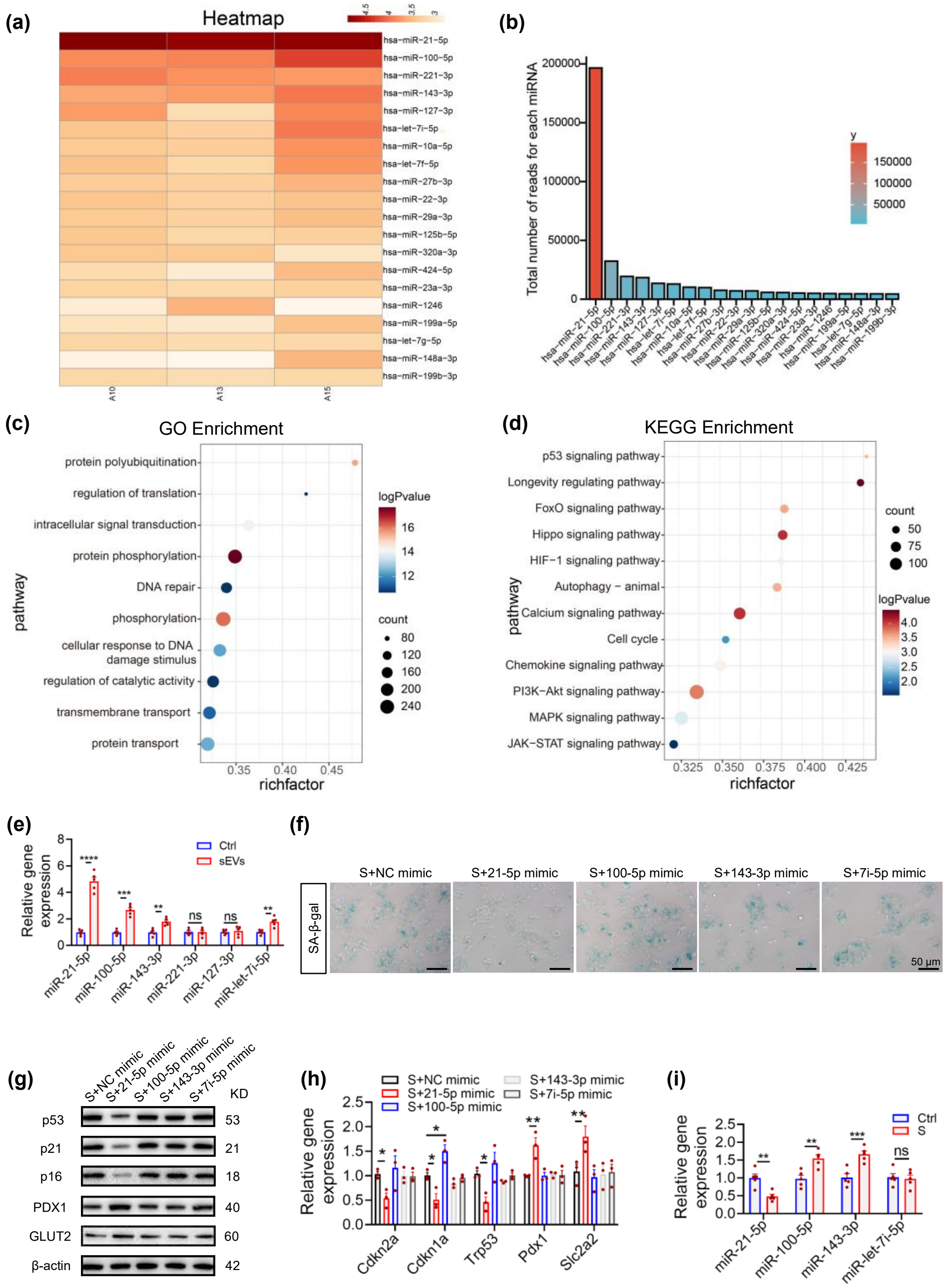

Supplementary Figure S6. miRNA cargo profiling and functional validation in hAMSC-sEVs. (a) Heatmap shows the top 20 highly expressed miRNAs in hAMSC-sEVs from three independent hAMSC lines (A10, A13, A15); expression is normalized as reads per million (RPM). **(b)** Bar graph shows the same top 20 miRNAs. **(c-d)** GO and KEGG enrichment are performed for predicted targets of six highly enriched miRNAs. **(e)** qPCR validates the six candidate miRNAs in MIN6 cells after hAMSC-sEV treatment (48 h). n = 5 per group. **(f–h)** Functional effects of miRNA overexpression in senescent MIN6 cells: **(f)** SA-β-gal staining; Scale bars, 50

µm; **(g)** Western blots show senescence markers (p53, p21, p16) and maturation markers (PDX1, GLUT2); **(h)** qPCR assesses *Cdkn2a, Cdkn1a, Trp53, Pdx1*, and *Slc2a2*. n = 3 per group. **(i)** qPCR quantifies four candidate miRNAs in senescent MIN6 cells. n = 5 per group. SA-β-gal staining is repeated in ≥2 independent experiments with similar results. Data are presented as mean ± SEM; ns, not significant; $^{*}P < 0.05$, $^{**}P < 0.01$, $^{***}P < 0.001$, $^{****}P < 0.0001$.

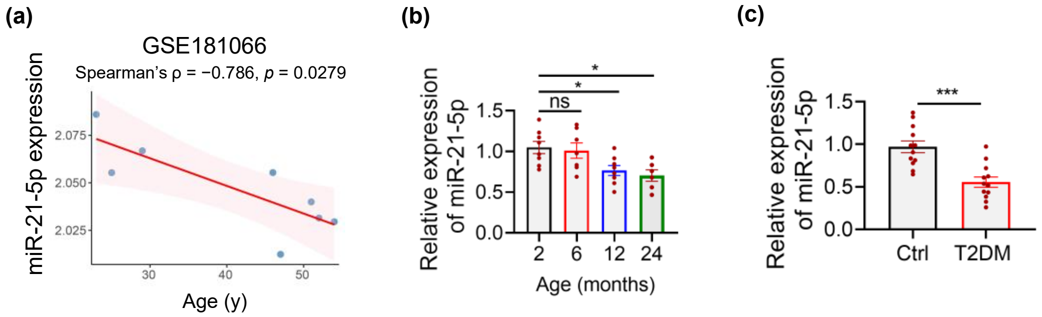

Supplementary Figure S7. Cross-species reduction of miR-21-5p in aging human β cells and mouse islets. (a) Human islets (GSE181066): Donor age is inversely correlated with β-cell miR-21-5p expression (Spearman's ρ = −0.786, p = 0.0279; n = 8 donors). **(b)** Naturally aged mice: RT–qPCR of isolated islets shows reduced miR-21-5p in aged versus young controls (n = 6–8 per group; normalized to U6). **(c)** Aged T2DM mice: RT–qPCR of islets shows reduced miR-21-5p relative to age-matched non-diabetic controls (n = 12 per group; each dot represents one mouse; normalized to U6). Data are presented as mean ± SEM; ns, not significant; *$P < 0.05$, ***$P < 0.001$.

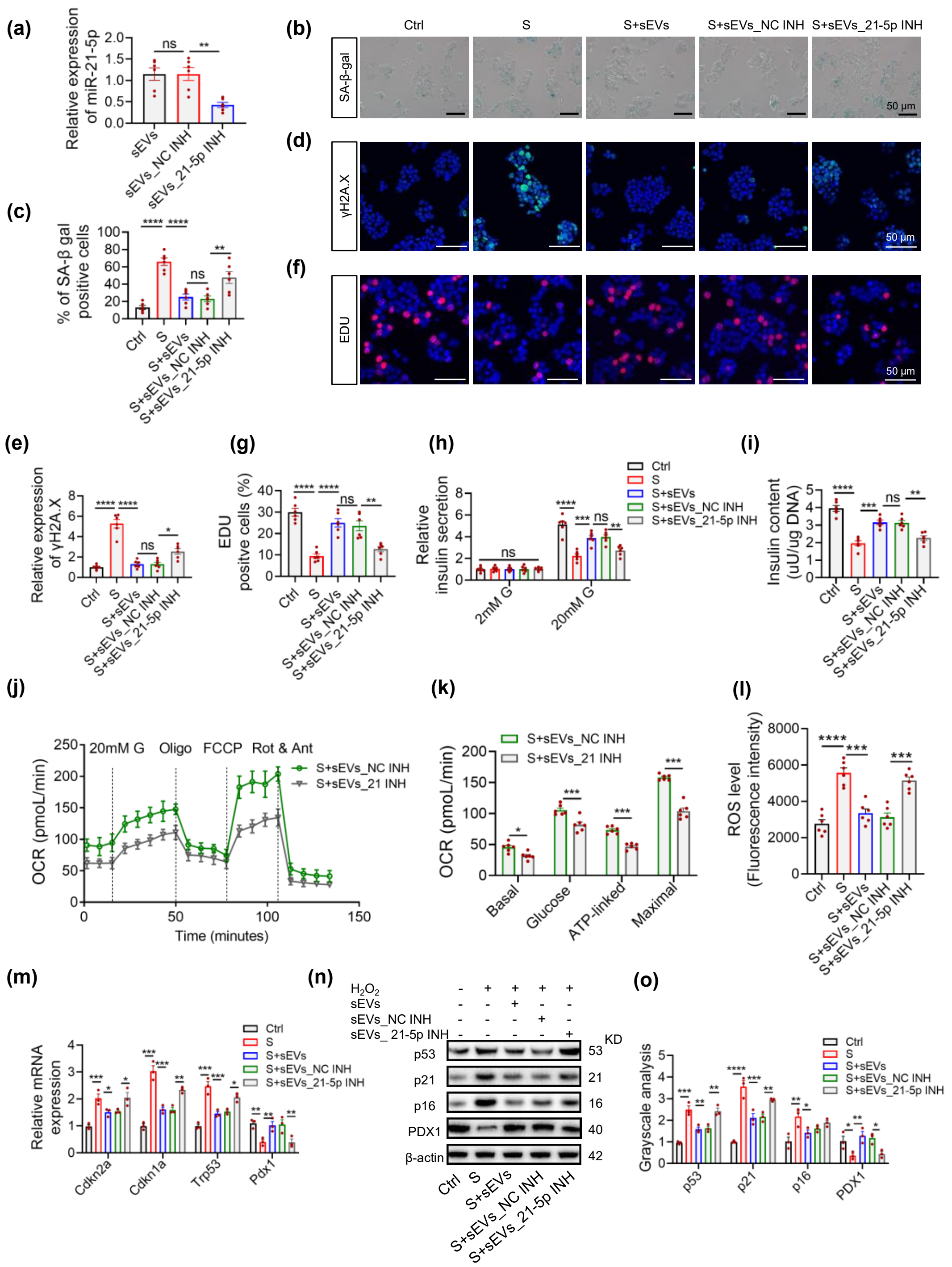

Supplementary Figure S8. miR-21-5p mediates hAMSC-sEV-driven senescent β-cell rejuvenation. (a) hAMSC-sEVs are loaded with a miR-21-5p inhibitor (sEVs-21-5p INH) or scrambled control (sEVs-NC INH); knockdown efficacy is validated by qPCR (normalized to U6; n = 6 per group). **(b–l)** H_2O_2-induced senescent MIN6 cells (200 μM, 2 h) are treated with native or modified sEVs (100 ng/μL, 48 h). **(b)** Representative SA-β-gal staining. Scale bars, 50 μm; **(c)** Quantification of SA-β-gal^{+} cells for (b); n = 6 per group. **(d)** Representative γ-H2AX immunofluorescence (IF). Scale bars, 50 μm; **(e)** Quantification of γ-H2AX IF intensity for (d); n = 6 per group. **(f)** Representative EdU IF. Scale bars, 50 μm; **(g)**

Quantification of EdU^{+} cells for (f); n = 6 per group. **(h)** GSIS; n = 6 per group. **(i)** Intracellular insulin content; n = 6 per group. **(j–k)** Oxygen consumption rate (OCR) is analyzed in S + sEVs-21-5p INH versus S + sEVs-NC INH; n = 6 per group. **(l)** ROS levels are measured under Ctrl, S, S + sEVs, S + sEVs-NC INH, and S + sEVs-21-5p INH; n = 6 per group. **(m)** qPCR assesses Cdkn2a, Cdkn1a, Trp53, and Pdx1; n = 3 per group. **(n)** Western blots show p53, p21, p16, and PDX1. **(o)** Densitometry quantifies bands in (n); n = 3 per group. SA-β-gal staining and immunofluorescence are repeated in ≥2 independent experiments with similar results. Data are presented as mean ± SEM; ns, not significant; $^{*}P < 0.05$, $^{**}P < 0.01$, $^{***}P < 0.001$, $^{****}P < 0.0001$.

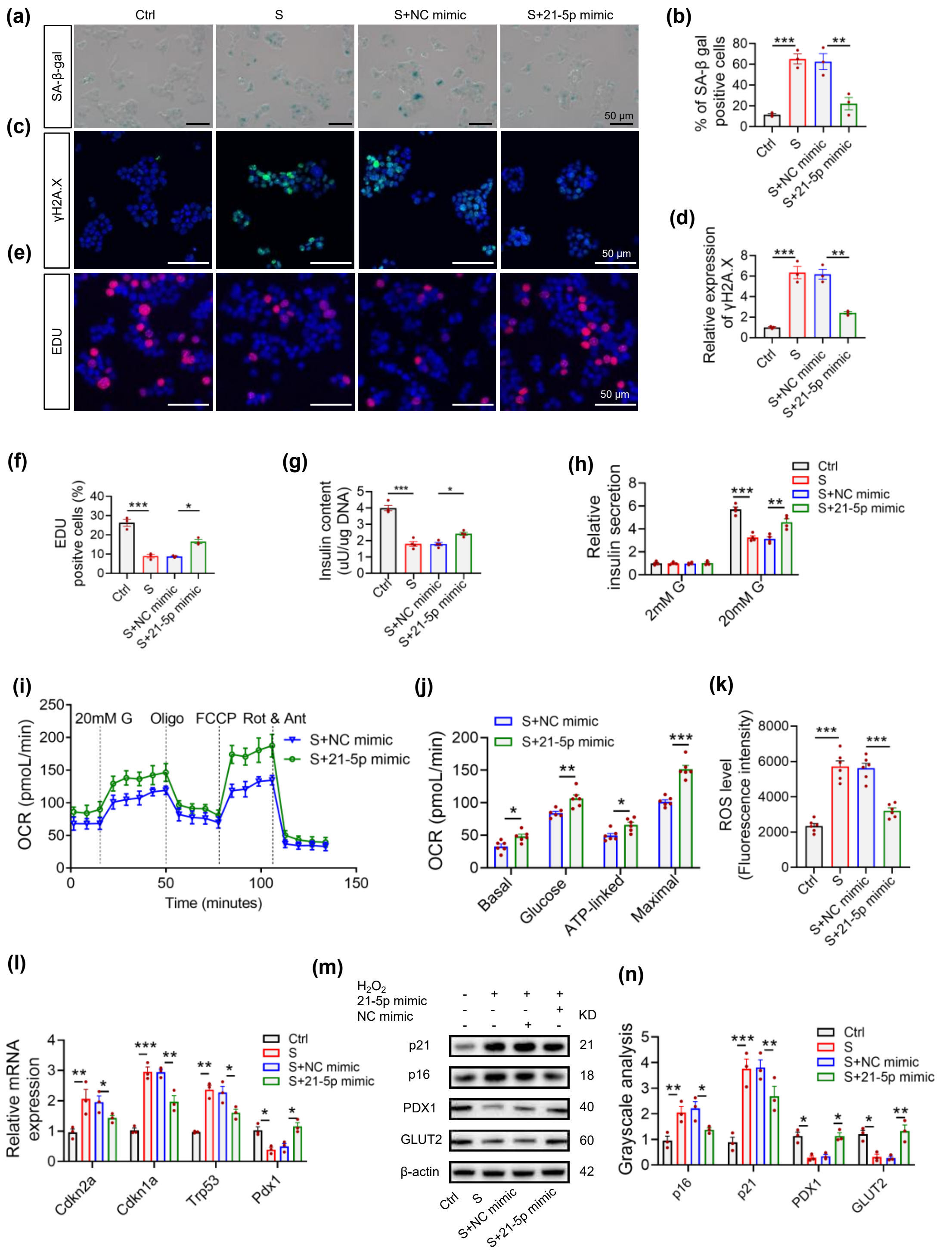

Supplementary Figure S9. miR-21-5p mimics the role of hAMSC-sEVs in anti-β-cell senescence. (a–h) H_2O_2-induced senescent MIN6 cells (200 μM, 2 h) are transfected with a miR-21-5p mimic (S + 21-5p mimic) or scrambled control (S + NC mimic); untreated senescent cells (S) and normal MIN6 (Ctrl) serve as controls. Phenotypic analyses are conducted 48 h post-transfection. **(a)** Representative SA-β-gal staining. Scale bars, 50 μm. **(b)** Quantification of SA-β-gal$^+$ cells; *n* = 3 per group. **(c)** Representative γ-H2AX immunofluorescence (IF). Scale bars, 50 μm. **(d)** Quantification of γ-H2AX IF intensity; *n* = 3 per group. **(e)** Representative EdU IF.

Scale bars, 50 µm**.** **(f)** Quantification of EdU$^+$ cells; n = 3 per group. **(g)** Intracellular insulin content; n = 4 per group. **(h)** GSIS; n = 4 per group. **(i–j)** Oxygen consumption rate (OCR) is analyzed in S + NC mimic and S + 21-5p mimic conditions; n = 6 per group. **(k)** ROS levels are measured in Ctrl, S, S + sEVs, S + NC mimic, and S + 21-5p mimic; n = 6 per group. **(l)** qPCR assesses *Cdkn2a, Cdkn1a, Trp53,* and *Pdx1* expression; n = 3 per group. **(m)** Western blots show p21, p16, PDX1, and GLUT2. **(n)** Densitometry quantifies bands in (m); n = 3 per group. SA-β-gal staining and immunofluorescence are repeated in ≥2 independent experiments with similar results. Data are presented as mean ± SEM; $^*P < 0.05$, $^{**}P < 0.01$, $^{***}P < 0.001$.

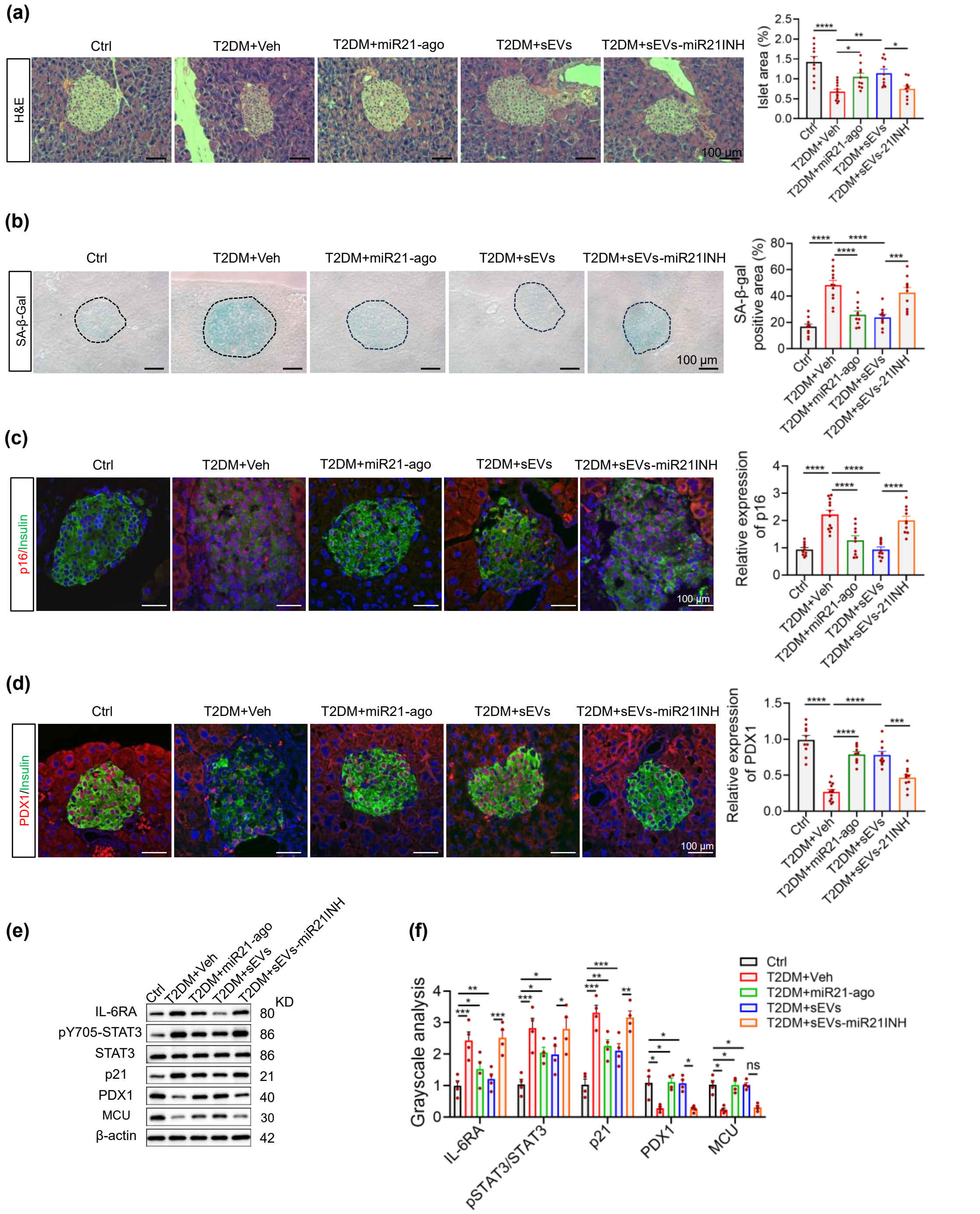

Supplementary Figure S10. miR-21-5p underlies hAMSC-sEV-mediated β-cell rejuvenation in aged diabetic mice. Eighteen-month-old C57BL/6J mice are rendered diabetic by HFD plus low-dose STZ and are treated for 8 weeks with vehicle, miR-21-5p agomir (10 mg/kg, i.v., once weekly), hAMSC-sEVs (1 mg/kg, i.v., twice weekly), or miR-21-5p–depleted sEVs (sEVs-21INH; 1 mg/kg, i.v., twice weekly); non-diabetic Ctrl is included. **(a)** H&E staining of pancreas with quantification of islet area (%). **(b)** SA-β-gal staining of islets with quantification of SA-β-gal–positive area (%) (islets outlined by dashed lines). **(c)** Immunofluorescence (IF) for p16 (red) and insulin (green) with Hoechst (blue) and quantification of β-cell p16. **(d)** IF for PDX1 (red) and insulin (green) with Hoechst (blue) and quantification of β-cell PDX1. **(e)**

Representative immunoblots of IL-6RA, pY705-STAT3, total STAT3, p21, PDX1, and MCU in islet lysates (β-actin loading control). **(f)** Densitometry: IL-6RA/β-actin, pSTAT3/STAT3, p21/β-actin, PDX1/β-actin, and MCU/β-actin. For IF quantification, each dot represents the per-mouse mean derived from ⩾15 islets per mouse sampled across ⩾3 non-adjacent sections; n = 10-12 mice per group. Bars indicate mean ± SEM. Scale bars, 100 μm. One-way ANOVA with Tukey's post hoc test (two-tailed); ns, not significant; *$P < 0.05$, **$P < 0.01$, ***$P < 0.001$, ****$P < 0.0001$.

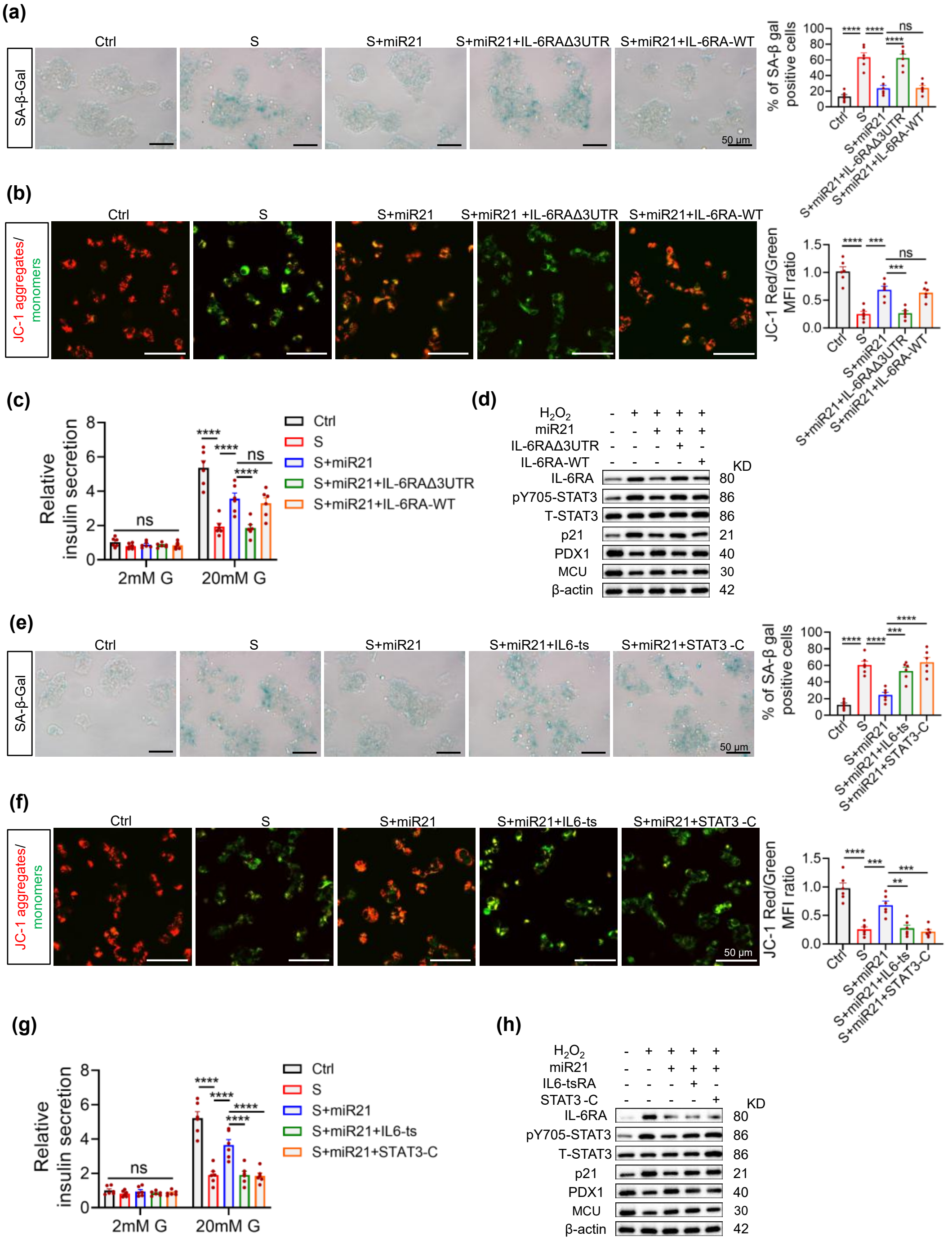

Supplementary Figure S11. miR-21-5p alleviates β-cell senescence by targeting the IL-6RA/STAT3 axis. (a) SA-β-gal staining for Ctrl, senescent (S; H_2O_2-induced), S + miR-21-5p (S + miR21), S + miR21 + IL-6RAΔ3′UTR (IL-6RA expression construct lacking the native 3′ UTR), and S + miR21 + IL-6RA-WT (full-length IL-6RA with native 3′ UTR). Right: % SA-β-gal^{+} cells. **(b)** JC-1 imaging for the same groups; mitochondrial depolarization under senescence is improved by miR-21-5p and reversed by IL-6RAΔ3′UTR. Right: JC-1 red/green MFI ratio. **(c)** GSIS at 2 mM and 20 mM glucose; miR-21-5p restores insulin secretion, which is blunted by IL-6RAΔ3′UTR but not IL-6RA-WT. **(d)** Immunoblots show decreased IL-6RA and pY705-STAT3 and restoration of PDX1 and MCU by miR-21-5p; IL-6RAΔ3′UTR

counteracts these effects. **(e)** SA-β-gal staining for Ctrl, S, S + miR21, S + miR21 + IL-6-ts (IL-6 + sIL-6R; STAT3 trans-signaling), and S + miR21 + STAT3-C (constitutively active STAT3). Right: quantification. **(f)** JC-1 imaging for the same groups; the miR-21-5p–rescued mitochondrial potential is abolished by IL-6-ts or STAT3-C. Right: JC-1 red/green MFI ratio. **(g)** GSIS; constitutive STAT3 activation (IL-6-ts or STAT3-C) antagonizes the pro-secretory effect of miR-21-5p. **(h)** Immunoblots show that IL-6-ts/STAT3-C restore pY705-STAT3 and p21 and suppress MCU and PDX1, opposing miR-21-5p. Data are presented as mean ± SEM (per-group *n* indicated in panels). One-way ANOVA with Tukey's post hoc test (two-tailed); ns, not significant; $^{**}P < 0.01$, $^{***}P < 0.001$, $^{****}P < 0.0001$. Scale bars, 50 μm.

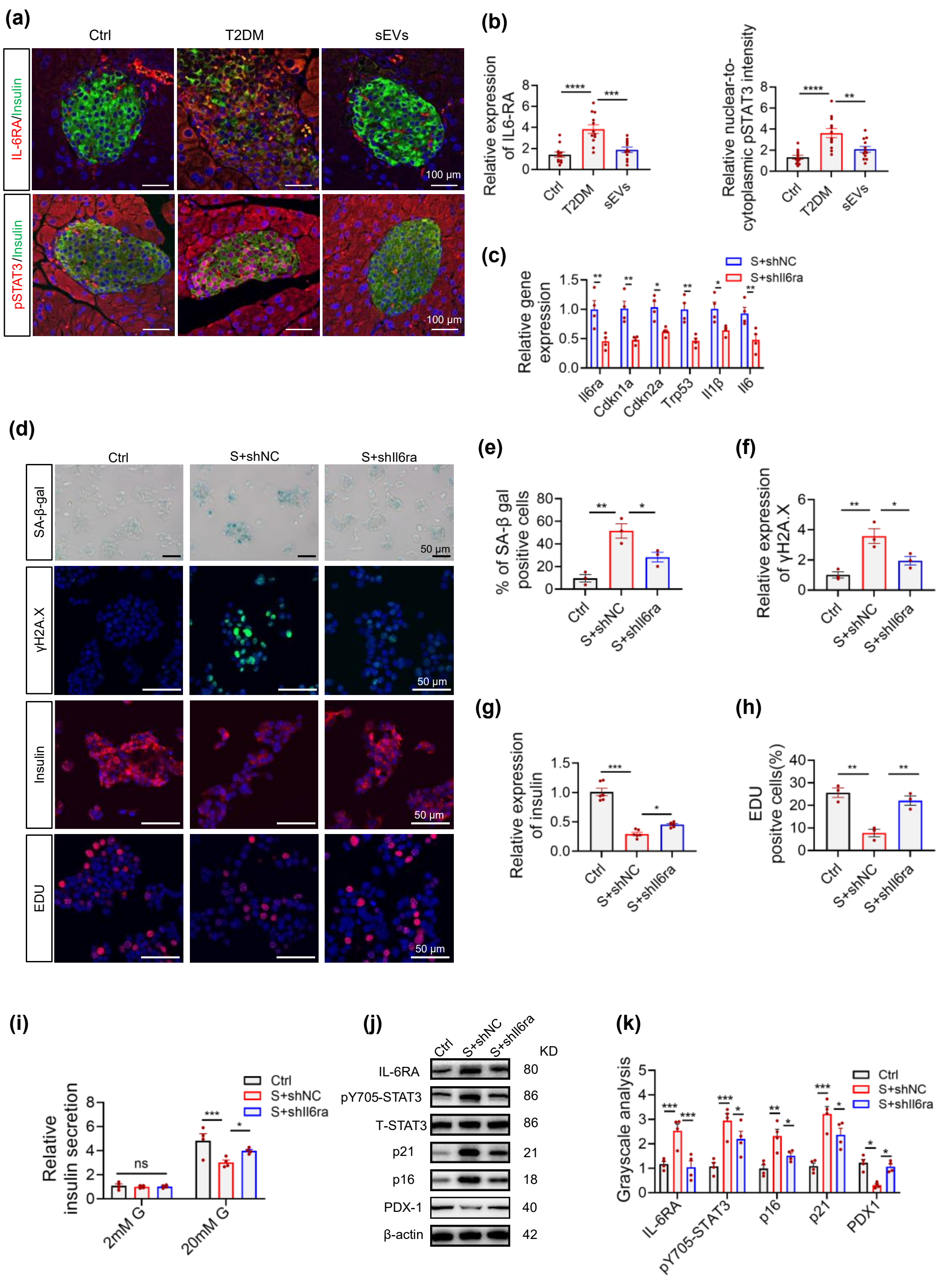

Supplementary Figure S12. Alleviating β-cell senescence via IL-6RA: hAMSC-sEVs downregulate IL-6RA and knockdown rejuvenates β-cells. (a-b) Immunofluorescence (IF) in pancreatic islets from Ctrl, T2DM, and T2DM + sEVs mice: insulin (green) with IL-6RA (red) and pY705-STAT3 (red) in (a), and quantification of β-cell IL-6RA IF and nuclear pSTAT3 (b). n = 12 mice per group; each dot represents the per-mouse mean from ≥15 islets sampled across ≥3 non-adjacent sections. Scale bars, 100 μm. **(c)** RT-qPCR profiling of senescence-associated genes in H_2O_2-induced senescent MIN6 cells transfected with

Il6ra shRNA (shIl6ra) versus scrambled control (shNC); n = 4 per group. **(d-k)** Phenotypic analyses 48 h after transfection of senescent MIN6 cells with shIl6ra or shNC; untreated normal MIN6 (Ctrl) serves as baseline. **(d)** Representative images of SA-β-gal (blue), γ-H2AX foci (green), insulin (red), and EdU incorporation (red). Scale bars, 50 μm. **(e–h)** Quantification of SA-β-gal$^+$ cells (%) **(e)**, γ-H2AX intensity **(f)**, insulin IF intensity **(g)**, and EdU$^+$ cells (%) **(h)**; n = 3-6 per group. **(i)** GSIS; n = 4 per group. **(j)** Immunoblots showing IL-6RA, pY705-STAT3, total STAT3, p16, p21, and PDX1. **(k)** Densitometry for (j); n = 4 per group. Data are presented as mean ± SEM; ns, not significant; $^{**}P < 0.01$, $^{***}P < 0.001$, $^{****}P < 0.0001$.

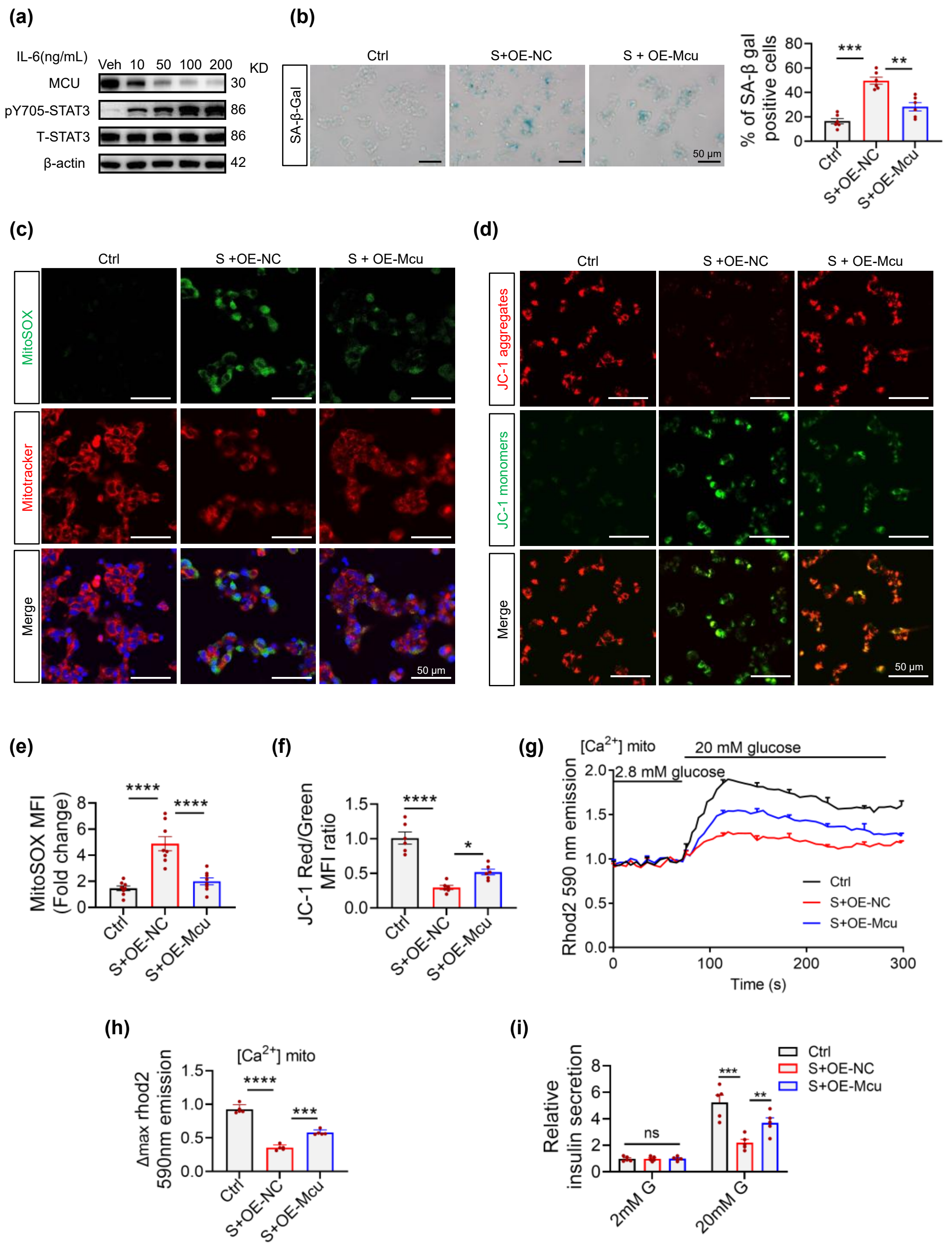

Supplementary Figure S13. Mcu overexpression exacerbates H_2O_2-induced mitochondrial dysfunction and cellular senescence in MIN6 cells. (a) Western blots show STAT3 phosphorylation (pY705-STAT3/total STAT3) and MCU expression in IL-6-treated MIN6 cells. **(b–i)** H_2O_2-induced senescence model (200 μM, 2 h); cells are transfected with OE-Mcu or OE-NC for 48 h. **(b)** Representative SA-β-gal staining with quantification of SA-β-gal^{+} cells; n = 6 per group. **(c)** Co-staining shows mitochondrial superoxide (MitoSOX, green) and mitochondrial mass (MitoTracker, red). **(d)** JC-1 staining indicates mitochondrial membrane potential (ΔΨm): red = high-ΔΨm aggregates; green = low-ΔΨm monomers. **(e)** Quantification of MitoSOX fluorescence for (c); n = 8 per group. **(f)** Quantification of

JC-1 red/green ratio (ΔΨm index) for (d); $n = 6$ per group. **(g-h)** Mitochondrial Ca^{2+} levels ($[Ca^{2+}]_{mito}$) are measured with Rhod-2 after 20 mM glucose stimulation; (g) shows average traces, (h) shows maximal Rhod-2 signals (normalized to basal); $n = 5$ per group. **(i)** GSIS; $n = 5$ per group. Data are presented as mean ± SEM; ns, not significant; $*P < 0.05$, $**P < 0.01$, $***P < 0.001$, $****P < 0.0001$. Scale bars, 100 μm. OE-Mcu, *Mcu*-overexpressing cells; OE-NC, vector control.

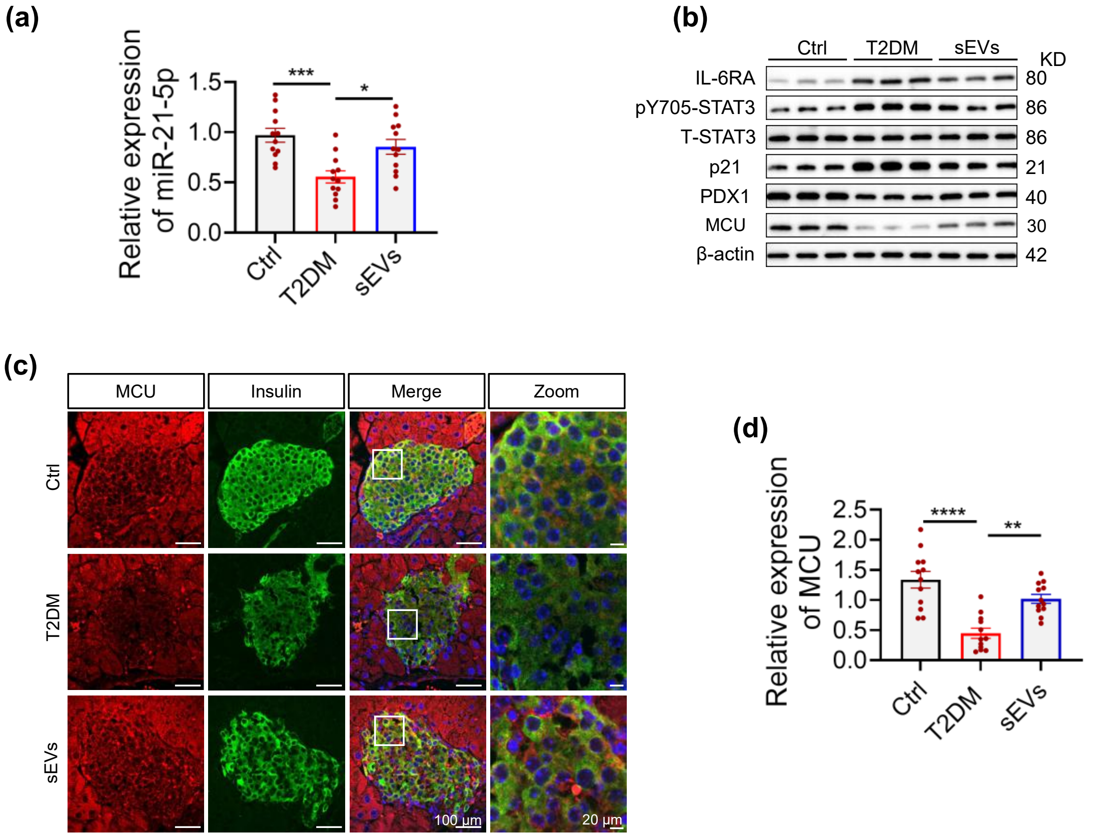

Supplementary Figure S14. hAMSC-sEVs restore the islet miR-21-5p/IL-6RA/STAT3/MCU axis in aged diabetic mice. (a) RT-qPCR shows miR-21-5p levels in isolated islets from Ctrl, T2DM, and T2DM + sEVs mice (normalized to U6; expressed relative to Ctrl). n = 12 mice per group. **(b)** Immunoblots show IL-6RA, pY705-STAT3, total STAT3, p21, PDX1, and MCU in islet lysates (β-actin loading control). **(c)** MCU immunofluorescence (red) with insulin co-staining (green); Merge and Zoom panels are shown. **(d)** Quantification shows β-cell MCU mean fluorescence intensity (MFI). n = 12 mice per group. Each dot represents one mouse. For IF quantification, per-mouse means are derived from ≥15 islets sampled across ≥3 non-adjacent sections. Data are presented as mean ± SEM; *$P < 0.05$, **$P < 0.01$, ***$P < 0.001$, ****$P < 0.0001$. Scale bars: 100 μm (overview panels); 20 μm (Zoom).
